# Tomato yellow leaf curl virus intergenic siRNAs target a host long noncoding RNA to modulate disease symptoms

**DOI:** 10.1371/journal.ppat.1007534

**Published:** 2019-01-22

**Authors:** Yuwen Yang, Tingli Liu, Danyu Shen, Jinyan Wang, Xitie Ling, Zhongze Hu, Tianzi Chen, Jieli Hu, Junyu Huang, Wengui Yu, Daolong Dou, Ming-Bo Wang, Baolong Zhang

**Affiliations:** 1 Provincial Key Laboratory of Agrobiology, Jiangsu Academy of Agricultural Sciences, Nanjing, China; 2 Department of Plant Pathology, Nanjing Agricultural University, Nanjing, China; 3 CSIRO Plant Industry, Canberra, Australia; University of California, Davis Genome Center, UNITED STATES

## Abstract

*Tomato yellow leaf curl virus* (TYLCV) and its related begomoviruses cause fast-spreading diseases in tomato worldwide. How this virus induces diseases remains largely unclear. Here we report a noncoding RNA-mediated model to elucidate the molecular mechanisms of TYLCV-tomato interaction and disease development. The circular ssDNA genome of TYLCV contains a noncoding intergenic region (IR), which is known to mediate viral DNA replication and transcription in host cells, but has not been reported to contribute directly to viral disease development. We demonstrate that the IR is transcribed in dual orientations during plant infection and confers abnormal phenotypes in tomato independently of protein-coding regions of the viral genome. We show that the IR sequence has a 25-nt segment that is almost perfectly complementary to a long noncoding RNA (lncRNA, designated as *SlLNR1*) in TYLCV-susceptible tomato cultivars but not in resistant cultivars which contains a 14-nt deletion in the 25-nt region. Consequently, we show that viral small-interfering RNAs (vsRNAs) derived from the 25-nt IR sequence induces silencing of *SlLNR1* in susceptible tomato plants but not resistant plants, and this *SlLNR1* downregulation is associated with stunted and curled leaf phenotypes reminiscent of TYLCV symptoms. These results suggest that the lncRNA interacts with the IR-derived vsRNAs to control disease development during TYLCV infection. Consistent with its possible function in virus disease development, over-expression of *SlLNR1* in tomato reduces the accumulation of TYLCV. Furthermore, gene silencing of the *SlLNR1* in the tomato plants induced TYLCV-like leaf phenotypes without viral infection. Our results uncover a previously unknown interaction between vsRNAs and host lncRNA, and provide a plausible model for TYLCV-induced diseases and host antiviral immunity, which would help to develop effective strategies for the control of this important viral pathogen.

## Author summary

*Tomato yellow leaf curl virus* (TYLCV) is an important threat to tomato production worldwide. Six resistance/tolerance genes (*Ty*) have been introgressed from wild tomato species for breeding against the virus and some of them confer tolerance to TYLCV by enhancing gene silencing. Here, we propose a novel model for TYLCV-induced symptoms and host antiviral immunity, which is independent of the known *Ty* genes. We show that the virus-derived vsRNAs may cause abnormal phenotypes similar to virus infection by targeting and regulating a lncRNA (designated as *SlLNR1*) in tomato. Furthermore, we find that *SlLNR1* contributes to the normal development and TYLCV resistance in tomato. Interestingly, *SlLNR1* exhibits a natural variant in a tomato cultivar that may evade vsRNAs-mediated cleavage. Thus, we suggest a novel evolutionary arms race between the vsRNAs and lncRNA, which could open new avenues in developing new viral control strategies.

## Introduction

TYLCV belongs to DNA geminivirus and can cause severe damages and yield loss to many important crops. Its infection results in stunted leaf growth and abnormal leaf development, including curling of leaf margins, reduction of leaf size, and yellowing and abscission of leaves. The disease symptoms and the virus were first reported in Israel during the late 1920s and the early 1960s, respectively [1, 2]. A great diversity of TYLCV has since been reported, including other five related species and numbers of strains. These viruses have been introduced in many areas due to international trade, which then adapted rapidly to the new environments and gave rise to new variants through recombination. The strong invasive capability of the viruses and absence of robust control practices contributed further to the worldwide spread and emergence of these viral diseases [3–5]. Understanding pathogenesis of TYLCV and its related species may help to develop innovative control strategies and answer some key questions regarding viral invasion and spread.

TYLCV has a circular single-stranded DNA genome which comprises a short intergenic region (IR) and six overlapping open reading frames (ORFs) in two opposite transcriptional directions [2]. It is widely believed that viral proteins encoded by these ORFs are pathogenicity determinants, and their biological activities and molecular functions during virus-host interactions have therefore been intensely studied [1, 6–8]. For instance, TYLCV AV2/V2 protein is found to be a multifunctional counter-defense factor, which suppresses host post-transcriptional gene silencing (PTGS) by targeting plant SGS3 protein, a dsRNA-binding protein, and then preventing SGS3 from accessing substrate RNAs [8, 9]. TYLCV V2 protein may also suppress transcriptional gene silencing (TGS) by reducing host DNA methylation [10]. In addition, V2 is shown to affect host cell death by interacting with and inhibiting enzymatic activity of CYP1, a plant programmed cell death machinery component [11]. A recent report showed that overexpression of V2 induced cell death symptoms in tomato, in contrast to no cell death caused by TYLCV infection [12]. But a healthy environment ensures the survival and multiplication of TYLCV, so the accumulation of TYLCV was hindered at the late stages of infection [12]. These results suggest that the individual viral proteins are insufficient to account for the TYLCV disease symptoms and other viral factor(s) may also contribute to the pathogenesis of the virus.

RNA silencing induced by double-stranded RNA (dsRNA) is an ancient mechanism in many eukaryotes that regulates gene expression and defends cells against invasive nucleic acids including viruses. During infection, virus derived dsRNA is processed by host Dicer-like enzymes to 21 to 24 nucleotide (nt) virus-derived small interfering RNAs (vsRNAs). These vsRNAs are loaded to argonaute protein to form RNA-induced silencing complex and guide the degradation of single-stranded viral RNA. As a counter-defense strategy, viruses encode RNA silencing suppressors to interfere with the vsRNA-directed silencing for successful infections [13–15]. These silencing suppressors are therefore major virulence determinants of viruses [16,17]. Some viruses have adapted another RNA silencing-based virulence strategy, using vsRNA-directed silencing of a host gene for symptom modification. *Cucumber mosaic virus* (CMV) is associated with a noncoding Y satellite RNA (Y-sat) that modifies yellowing symptoms in some hosts. There is a 22-nucleotide (nt) complementary sequence between Y-sat and tobacco magnesium protoporphyrin chelatase subunit I (*ChlI*, the key gene involved in chlorophyll synthesis) gene. During interactions, Y-sat- siRNAs derived from the 22-nt region target and cleave the host *ChlI* gene to impair the chlorophyll biosynthesis pathway and cause yellowing symptoms [18, 19]. Small RNA derived from the virulence modulating region of two *Potato spindle tuber viroid* variants target the callose synthase genes of tomato plants, thus causing leaf curling and severe stunting [20]. In *Botrytis cinerea*, a fungal pathogen, small RNAs also act as effectors to inhibit host immunity by silencing host immune genes [21]. It is unknown whether TYLCV and other viral pathogens employ vsRNAs as virulence effectors.

Plants have evolved additional layers of immune responses to combat viruses, and some of these immune processes intersect with RNA silencing. For instance, viral RNA silencing suppressors can be monitored by plant R proteins to initialize effector-triggered immunity (ETI) [16, 22, 23]. The P38 RNA silencing suppressor of *Turnip crinkle virus* and the 2b suppressor of *Tomato aspermy virus* have been shown to elicit hypersensitive response in tobacco [22] and in some specific *A*. *thaliana* ecotypes [23], respectively. Furthermore, RNA silencing pathways play a role in fine tuning *R* gene expression, by promoting gene expression levels or minimizing fitness cost of over-active virus resistance [24]. For TYLCV, six resistance/tolerance loci have been identified in tomato, including *Ty-1*, *-3*, -*4*, and *-6* from *Solanum chilense*, *Ty-2* from *S*. *habrochaites*, and *Ty-5* from *S*. *peruvianum*. All these six *ty* genes confer a tolerance phenotype allowing low levels of virus replication [25]. Among them, *Ty-2* gene was identified as an NBS-LRR gene, *TYNBS1* [26]; *Ty-1* and *Ty-3* genes code for an RNA-dependent RNA polymerase, and provide resistance by increasing cytosine methylation of the viral genome causing transcriptional gene repression of viral genes [27]. Thus, small RNA-directed RNA silencing therefore plays an important role in the molecular arm race between host plants and viruses, including TYLCV.

Besides small RNAs, lncRNAs have emerged as new RNA regulators of gene expression in eukaryotes through diverse molecular mechanisms [28, 29]. Many lncRNAs were shown to be associated with defense responses against biotic stresses, although their roles in plant-virus interactions are not reported. A number of *Puccinia striiformis*- and *Fusarium oxysporum*- responsive lncRNAs were identified in wheat and *Arabidopsis thaliana* [30, 31], respectively, five of which have important roles in plant defense against *F*.*oxysporum* infection [30]. An elf18 induced lncRNA *ELENA1* confers resistance to *Pseudomonas syringe* pv tomato DC3000 through interacting with Mediator subunit 19a (MED19a) and affecting its enrichment on the *PR1* promoter [32]. Tomato lncRNA16397 appears to play a role in plant resistance to late blight disease by regulating the expression of *SlGRX*, a gene family in plant reactive oxygen species (ROS) scavenging systems [33]. Overexpression of lncRNA16397 reduces ROS accumulation and alleviates cell membrane injury [33]. On the contrary, some lncRNAs were reported facilitating the disease infection such as the *GhlncNAT-ANX2* and *GhlncNAT-RLP7* to *Verticillium dahlia* of cotton [34]. Our previous research has characterized tomato lncRNAs during its interactions with TYLCV and found that some lncRNAs might act as small RNA target mimics to participate in the regulatory process of tomato viral resistance [35].

Here we report a direct interaction between vsRNAs and host-encoded lncRNA and its involvement in TYLCV disease development in tomato. We discovered that a 25-nt short segment of the TYLCV intergenic region (IR) has near-perfect complementarity with a tomato lncRNA, termed as *SlLNR1*, and IR-derived vsRNAs direct silencing of the lncRNA during viral infection. We showed that *SlLNR1* plays a role in TYLCV resistance and leaf development in tomato, and downregulation of the lncRNA by IR-derived vsRNAs results in TYLCV-like symptoms. Intriguingly, its allele in a TYLCV-tolerant has a 14-nt sequence deletion, making it resistant to vsRNA-directed repression. Our findings suggest that TYLCV deploys IR-derived vsRNAs as virulence effector to interfere with *SlLNR1* and induce the stunted and curled leaf phenotypes of TYLCV symptoms. Our study provides a viral disease model involving an arm race between viral small RNAs and a host lncRNA.

## Results

### The IR can be bidirectionally transcribed and cause abnormal phenotypes in TYLCV-susceptible tomato cultivar

**Fig 1:**
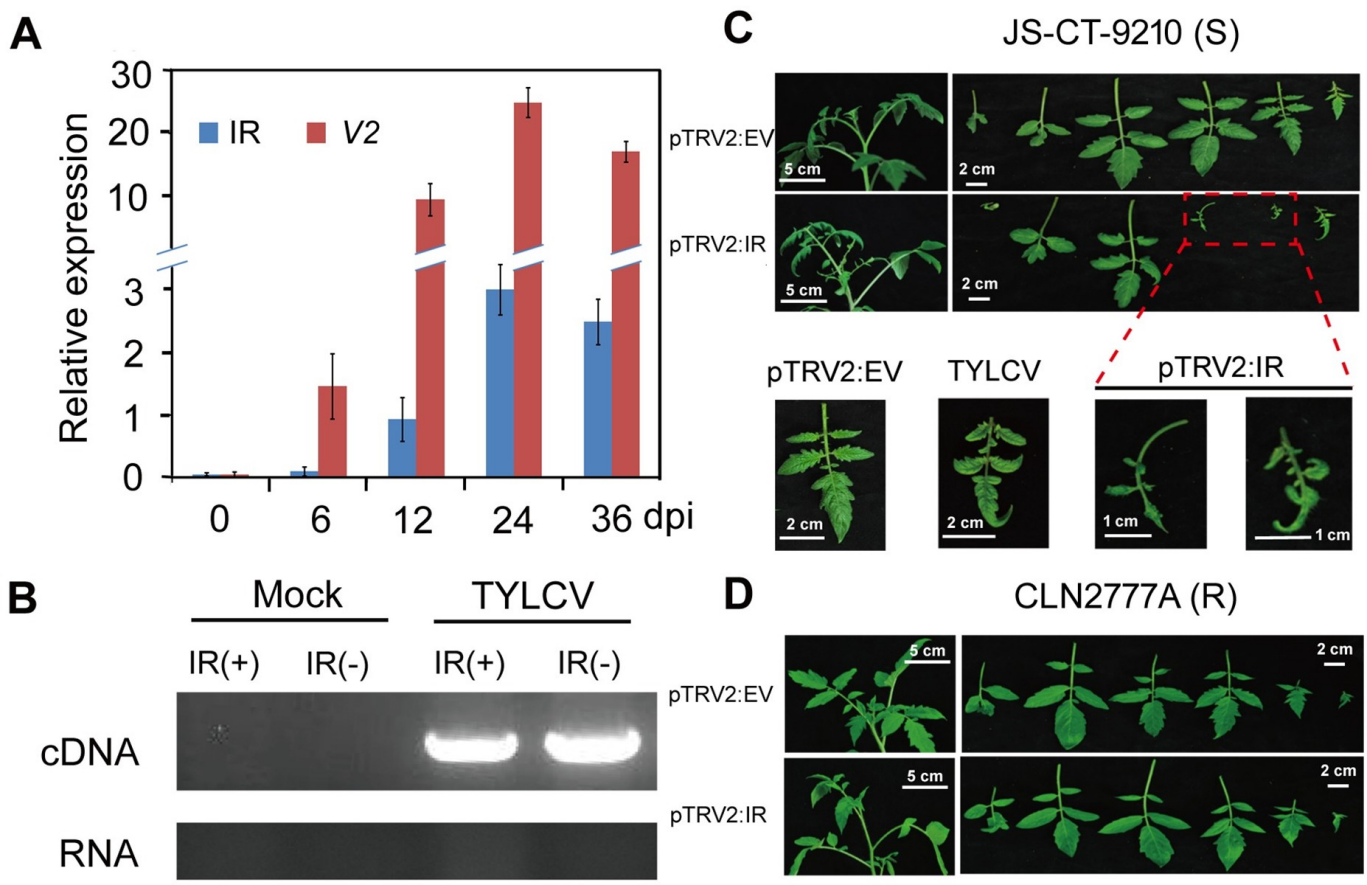
The TYLCV IR is bidirectionally expressed during infection and confers abnormal phenotypes in tomato. **(A)** Accumulation of TYLCV IR and *V2/AV2* transcripts. The generated TYLCV-infectious clone was inoculated on tomato seedling at the 3 to 4-true leaf stages. The samples were collected at the indicated days post inoculation (dpi) and used for RNA extraction. The transcript levels of IR and *V2* were normalized to tomato *actin* gene. Error bars represented SE of three biological replicates. **(B)** The IR is transcribed in both directions. The IR transcript could be amplified from the cDNA transcribed with sense- or anti-sense-specific primers in TYLCV infected plants, and no-RT RNA template was used as a negative control to validate no DNA contamination. Mock indicates the uninfected plants. **(C/D)** Phenotypes of TYLCV–susceptible (C) and–resistant (D) tomato lines. The plants were inoculated with EV (pTRV2:EV), pTRV2 containing IR (pTRV2:IR) and TYLCV infectious clone (TYLCV). The leaves were arrayed according to the order from the bottom to the top of tomato plants. The amplified images were shown as indicated at the lower panel. The photos were taken at 15 dpi.

Considering that the IR in geminivirus is a major source of vsRNAs [36–38], we examined whether the TYLCV IR could be transcribed. Indeed, tomato plants infected using a TYLCV infectious clone [39], contained an increasing levels of the IR transcripts during infection, despite the relatively lower abundance than the *V2* transcript (Fig 1A). The IR transcripts (nt 2616–147) can be amplified using both sense and antisense strand-specific primers in TYLCV infected plants while no visible amplicons were found in the negative controls (Fig 1B), indicating that the IR sequence is transcribed bidirectionally.

To examine if the IR transcript plays a role in viral virulence and symptom development, we inoculated a TYLCV-susceptible (JS-CT-9210) or -resistant (CLN2777A) tomato line with a tobacco rattle virus vector containing the IR sequence (nt 2616–147) (pTRV2:IR). As shown in Fig 1C, expression of IR from the TRV vector resulted in stunted and curled leaves and stems in the susceptible tomato genotype, which resemble the symptoms caused by TYLCV infection [39]. In contrast, its expression in the resistant tomato line failed to induce TYLCV-like symptoms (Fig 1D), although the transcriptional levels of IR were comparable in these two lines (S1A Fig). Similar to IR RNA levels, the viral accumulation of TRV was similar between the two lines as indicated by the similar amounts of TRV-specific vsRNAs (S1B Fig) and expressional levels of replicase and 2b-encoding genes (S1C Fig). This excluded the possibility that the different phenotypes between the susceptible and resistant tomato lines are caused by different TRV accumulation hence virulence (S1 Fig). Thus, we conclude that the IR alone is sufficient to induce symptoms in susceptible but not resistant tomato background, suggesting that it not only mediates TYLCV genome replication but also plays a direct role in viral virulence.

### siRNAs derived from IR can also cause abnormal phenotypes

**Fig 2:**
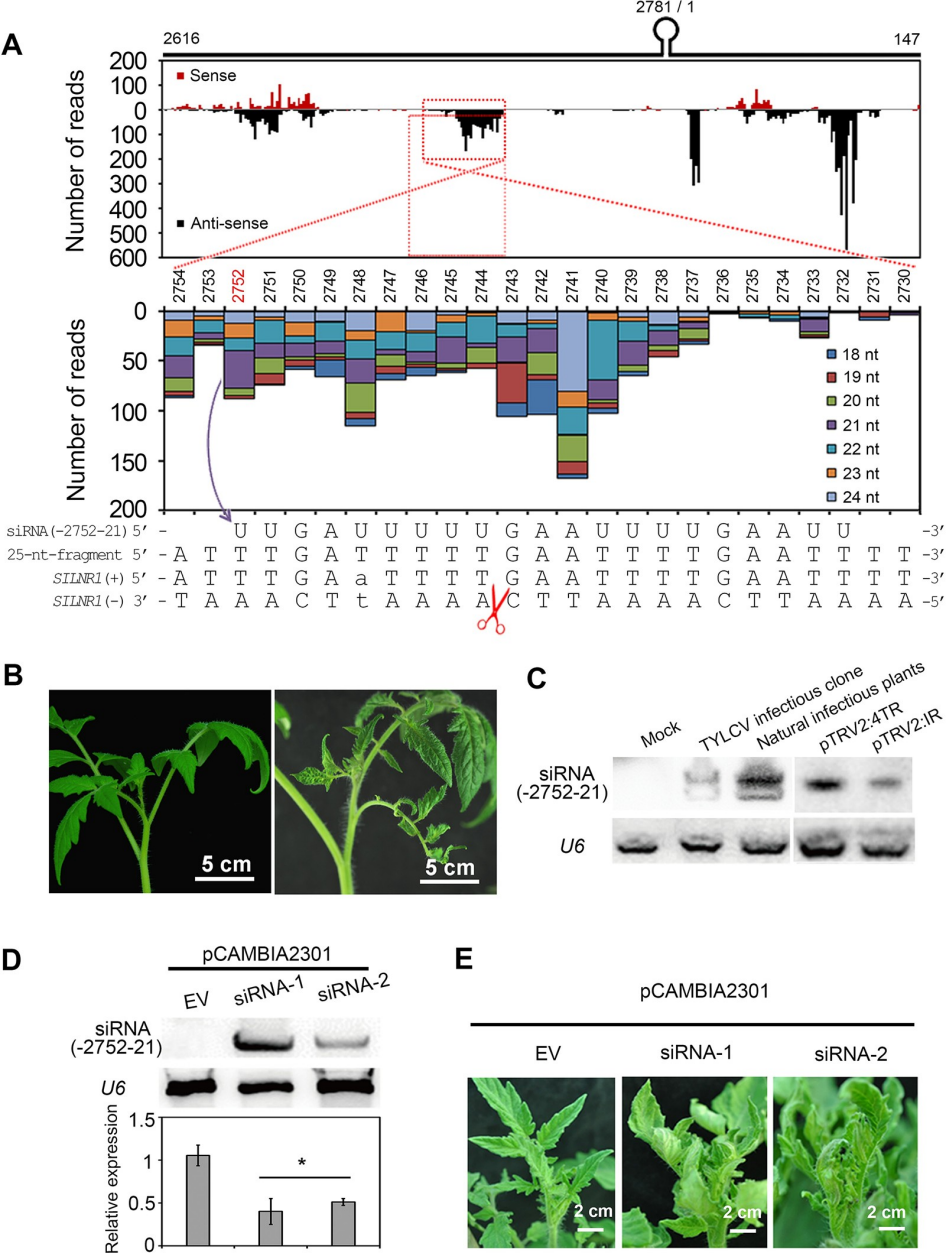
Identification of a 25-nt segment and a vsRNA that induce stunt and curled leaves in tomato. **(A)** VsRNAs generated by the IR and sequence alignment of vsRNAs and *SlLNR1*. Location and frequency of TYCLV-derived siRNAs (vsRNAs) were mapped to the IR in sense- (above the x-axis) or antisense- (below the x-axis) orientation. Genome organization of the IR was shown at the top in which the inverted repeat was symbolized as a stem loop. Numbers indicate the first (2616) and last (147) nucleotides of the IR sequence. The histogram of location, frequency and size distribution of vsRNAs corresponding to the 25-nt-fragment (2730–2754) were shown at the medium panel. The fragment was highly complemented with *SlLNR1*(-). The scissor means the cleavage site determined by 5’-RACE analysis. **(B)** Phenotypes of tomato inoculated with pTRV2:4TR. TYLCV-susceptible tomato plants were inoculated with pTRV2 containing 4×25-nt-fragment (2730–2754). The photos were taken at 15 dpi. **(C)** Validation of siRNA(-2752-21) in the tomato plants by siRNA Northern blot. The leaves of susceptible tomato plants inoculated by TYLCV infectious clone, natural infection by viruliferous whiteflies, agroinfiltrated with pTRV2:4TR and pTRV2:IR were used for total RNA extraction and analyzed at 24 dpi. *U6* gene was set as the internal control. **(D)** Validation of siRNA(-2752-21) presence and downregulation of *SlLNR1* in the overexpressed plants. Two individual transgenic lines (pCAMBIA2301:siRNA-1/2) with overexpression of siRNA(-2752-21) were used for total RNA extraction and analyzed. EV indicates the transgenic plant with the EV. The lower panel shows the relative expressi*o*n of *SlLNR1* that was measured by qRT-PCR and calculated in relation to the transgenic plants according to the ^ΔΔ^Ct method using tomat*o actin* gene as the reference. Error bars represented SE of three biological replicates and significant differences by Student’*s t* test (*, p<0.05) **(E)** Phenotypes of siRNA(-2752-21) overexpressed plants. The typical photos were taken at 60 days after seed germination.

The IR does not encode proteins, but gives rise to vsRNAs during viral infection. We therefore investigated whether IR-derived vsRNAs might be responsible for IR-induced symptoms by directing host gene silencing as reported for Cucumber mosaic virus Y-satellite-derived siRNAs [19, 40]. We firstly characterized vsRNAs in TYLCV-infected tomato plants using small RNA deep sequencing. Overall, vsRNAs were discontinuously and unequally distributed along the TYLCV genome (S2A Fig), which is consistent with the previous reports on other geminivirus-derived siRNAs [36–38]. IR-derived vsRNAs had a total of 49,569 reads (2.4% of all vsRNA reads) with 845 unique sequences. To identify potential host target genes of vsRNAs, we aligned the IR-derived vsRNAs to available tomato sequences using BLAST, and identified an EST sequence with 25-nt near-perfect complementarity with a 25-nt segment of IR (nt 2730–2754) that is associated with relatively high abundance of vsRNAs (Fig 2A). The tomato EST was predicted to encode no protein, and therefore named *SlLNR1* (*Solanum lycopersicum* lncRNA1). The high-level sequence complementarity raised the possibility that vsRNAs derived from the 25-nt IR segment induce silencing of the complementary tomato target RNA to mediate the IR-induced symptoms. Consistent with this, a pTRV2 vector carrying 4 tandem repeats of the 25-nt segment (pTRV2:4TR) was capable of inducing stunted and curled leave phenotypes (Fig 2B), similar to those caused by pTRV2:IR containing the IR sequence (Fig 1C). At the same time, we inoculated the above constructs in tobacco (a TYLCV host) and cotton (a nonhost of TYLCV), and found that both pTRV2:IR and pTRV2:4TR could not cause abnormal phenotypes (S2B Fig) although they were expressed properly (S2C Fig). Sequence similarity searching in other plants failed to find homologues of the *SlLNR1*. Taken collectively, the above results demonstrate that the IR-mediated virulent activities are unique in tomato.

To confirm the accumulation of the vsRNAs from the 25-nt IR segment during TYLCV infection, we performed siRNA northern blot to detect a 21-nt antisense siRNA (-2752-21), which was chosen because of its relatively high abundance in the deep sequencing data (Fig 2A). As shown in Fig 2C, this siRNA was detected in the plant inoculated by TYLCV infectious clone at 24 dpi. We also analyzed the tomato plants infected using viruliferous whiteflies in the green house, and this siRNA was also can be detected (Fig 2C). In addition, the siRNA is also expressed in the plants inoculated by pTRV2:4TR and pTRV2:IR (Fig 2C).

To examine the potential biological function of siRNA (-2752-21), its overexpression construct driven by the 35S promoter was transformed to susceptible tomato plants, and 2 lines of overexpressed plants (pCAMBIA2301:siRNA-1/2) were obtained. The plants were verified by accumulation of the object siRNA (Fig 2D), and exhibited abnormal phenotypes as stunted and curled leaves (Fig 2E). Taken together, the results from siRNA analyses and host target gene searching supported an involvement of vsRNA-directed host gene silencing in IR-induced symptom development, and suggested that vsRNAs from the 25-nt IR segment are the silencing inducer, and *SlLNR1* is likely a host target.

### *SlLNR1* is a target of IR-derived siRNA

**Fig 3:**
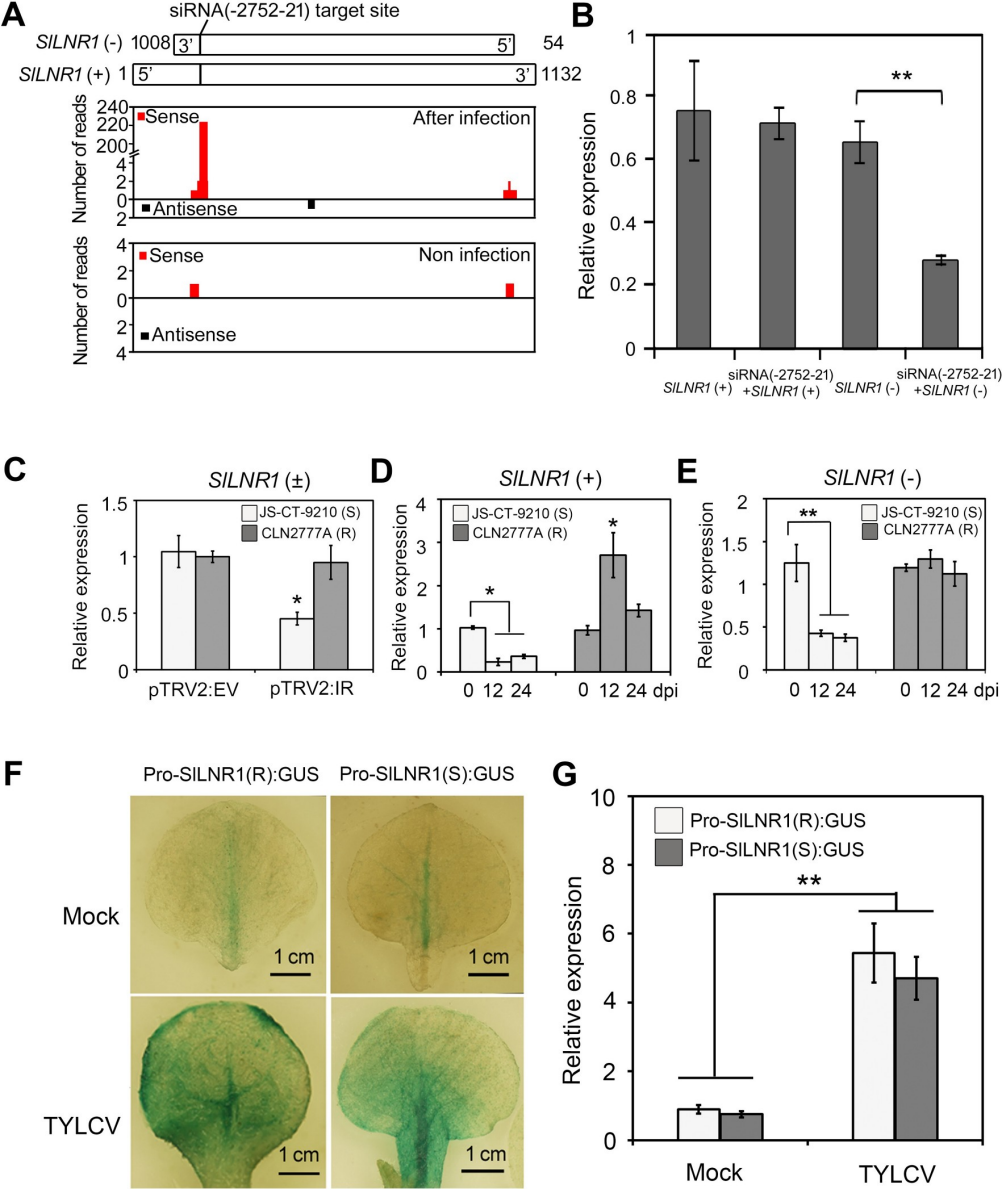
siRNA (-2752-21) targets tomato *SlLNR1*. **(A)** Schematic illustration of *SlLNR1* structure and its associated siRNAs. The sense *SlLNR1*, *SlLNR1*(+), from the susceptible cultivar is an 1132-nt lncRNA, which is validated by RACE. Its anti-sense, *SlLNR1*(-), is generated by sequencing a segmental RT-PCR (the 54^th^ bp of the 3’ end to the 1008^th^). The full sequence of *SlLNR1* was shown in S4 Fig. The vertical bar indicate the target site of siRNA(-2752-21). The data derived from small RNA sequencing of TYLCV infected plants or mock was aligned to *SlLNR1* and the number indicates the aligned siRNA reads. **(B)** Negative correlation of expression of siRNA(-2752-21) and *SlLNR1*. *SlLNR1*(+) or *SlLNR1*(-) was expressed with siRNA(-2752-21) in *N*. *benthamiana*. The RNA sample was extracted at 48 h after agroinfiltration. The *EF1a* gene of *N*. *benthamiana* was used as a refernce. **(C)** *SlLNR1* was down regulated in the pTRV2:IR inoculated susceptible plants but not in the resistant plants. The RNA sample was extracted at 15 days after pTRV2:IR and EV inoculated plants. The relative expression of *SlLNR1* was measured by qRT-PCR and calculated in relation to EV inoculated plants according to the ^ΔΔ^Ct method. The tomato *actin* gene was set as reference gene. Error bars represented SE of three biological replicates and significant differences by Student’s *t* test (*, p<0.05). **(D-E)** Expressional profiles of the *SlLNR1*(+) (D) and *SlLNR1*(-) (E) during TYLCV infection. The relative expression of *SlLNR1* was measured by qRT-PCR and calculated in relation to the non-inoculated samples at 0 dpi according to the ^ΔΔ^Ct method with *actin* as a reference gene. Error bars represented SE of three biological replicates and significant differences by Student’s *t* test (*, p<0.05; **, p<0.01). **(F)** Gus staining of tobacco leaves at 12 dpi of TYLCV infection and the mock plants. The promoter of *SlLNR1*(R/S) (1.5 kb upstream sequence) was fused with *GUS* genes and transformed into tobacco for GUS staining. (G) The relative GUS quantitation of the tobacco leaves at 12 dpi of TYLCV infection and the mock plants. The *EF1a* gene of tobacco was set as the reference. Error bars represented SD of three biological replicates and significant differences by Student’s *t* test (**, p<0.01).

The full length sequence of *SlLNR1* (*SlLNR1+*) was further validated by 5’ RNA Ligase Mediated Rapid amplification of cDNA ends (5’ RLM-RACE) and 3’ RACE-PCR (S3 Fig), based on which an 1132-nt sense strand of *SlLNR1* was obtained in the susceptible cultivar by RT-PCR. Meanwhile, a 955-nt antisense-strand of *SlLNR1* (*SlLNR1-*) was also recovered using strand-specific RT-PCR (S4 Fig), indicating that *SlLNR1* is bi-directionally transcribed. The *SlLNR1*-specific sRNAs could be detected in TYLCV infected susceptible tomato plants by sRNA deep sequencing, while which can hardly be detected of the control plants despite the existence of both sense and antisense RNA that can potentially form double-stranded RNA (dsRNA) (Fig 3A). Interestingly, the *SlLNR1* sequence from the resistant tomato cultivar contained a 14-nt deletion in the region matching the 25-nt IR segment (S4 Fig), which would prevent binding and targeting by the IR-derived vsRNAs. Thus, we surmised that vsRNAs may specially target antisense *SlLNR1* from the susceptible cultivar, but not the sense *SlLNR1* or the *SlLNR1* transcripts from the resistant cultivar due to lack of sequence complementarity. Consistent with this, the antisense *SlLNR1*, but not the sense *SlLNR1*, was markedly downregulated in *N*. *benthimiana* when co-expressed with siRNA(-2752-21) (Fig 3B).

In addition, *SlLNR1*-specific sRNA can also be detected in the tomato plants inoculated by pTRV2:IR while quite few associated reads were found in the EV-infiltrated plants (S5 Fig). Next, we surmised that vsRNAs may specially target the *SlLNR1* in the susceptible cultivar, but not in the resistant cultivar due to lack of sequence complementarity. Consistent with this, the transcripts of *SlLNR1* were downregulated in the pTRV2:IR inoculated susceptible but not resistant tomato line (Fig 3C). Furthermore, upon TYLCV infection, the *SlLNR1* expression levels were downregulated at 12 and 24 dpi, whereas in the resistant tomato cultivar the level of the *SlLNR1* transcripts either increased (for the sense transcript) or remained unchanged (for the antisense transcript) (Fig 3D and 3E). Importantly, we observed that the expression of *SlLNR1* was also downregulated in the siRNA(-2752-21)- overexpressed plants (Fig 2F).

Then, the promoter of *SlLNR1* (for the sense transcript) from the susceptible or resistant line was constructed to drive the GUS reporter gene expression in *N*. *tabacum*. The results of GUS staining indicated both the promoters can be induced by the TYLCV infection at 12 dpi (Fig 3F). And the expression of GUS was also increased at the same time (Fig 3G). These results confirmed that the promoters of both the cultivars can be induced by TYLCV, and the *SlLNR1* from the susceptible line could be silenced during infection but not in the resistant cultivar. The downregulation of the sense *SlLNR1* could be due to the existence of antisense vsRNAs from the 25-nt IR segment, as viral dsRNA is expected to be processed into both sense and antisense vsRNAs. The absence of these antisense vsRNAs in the deep sequencing data could be due to relatively low abundance or sequestration by long sense viral RNAs as reported previously [41, 42].

**Fig 4:**
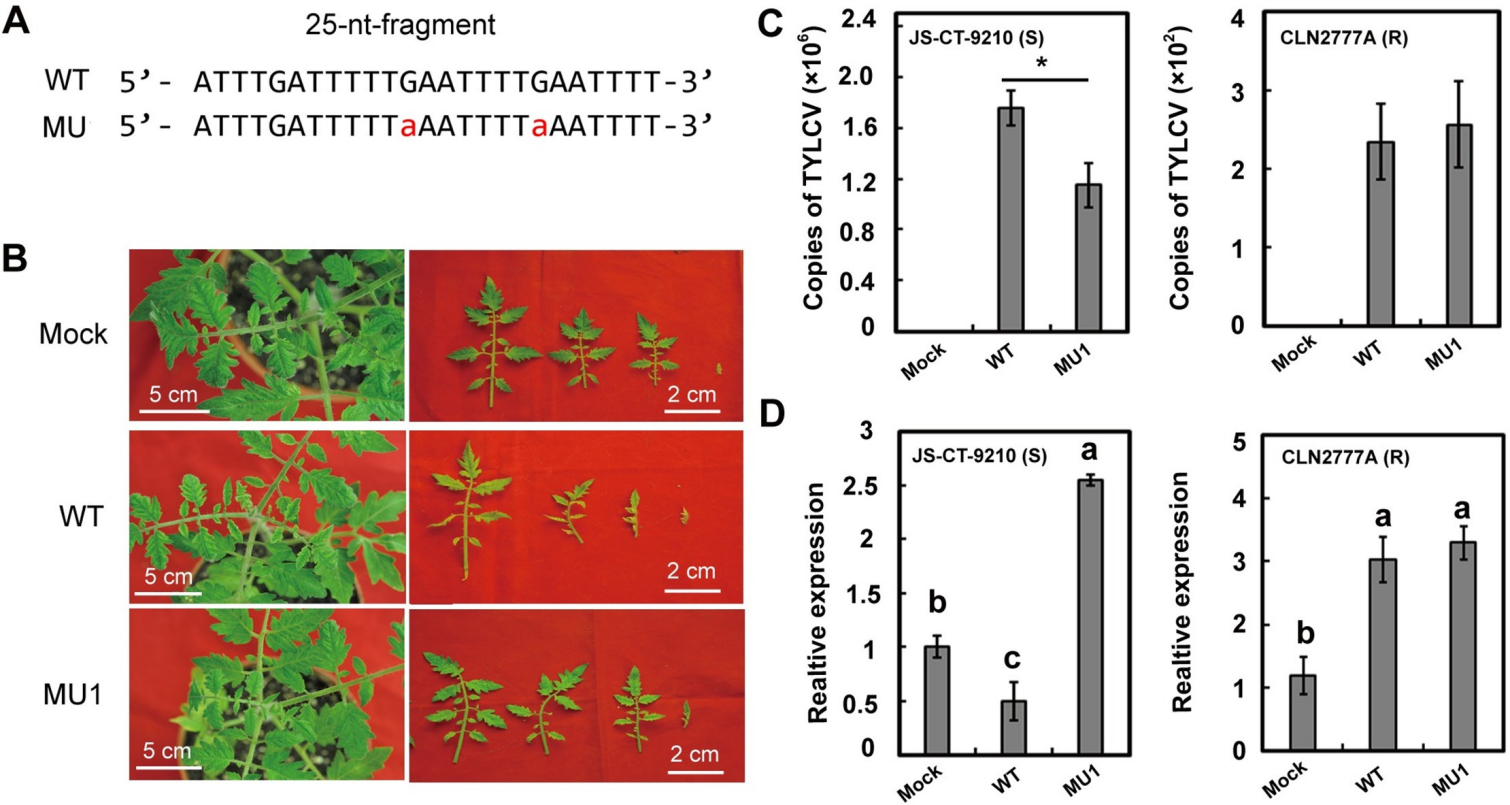
Sequence complementarity between siRNA(-2752-21) and the *SlLNR1* gene is important for the induction of pathogenetic phenotype. **(A)** Schematic diagram of the 25-nt fragment of IR and the corresponding mutant sequence in MU1. The mutant TYLCV infectious clone (MU1) was constructed with the mutated nucleotides indicated as the red letters. **(B)** The phenotype of the tomato plants. The photos were taken at 15 dpi. Mock indicates the tomato plants uninfected. The right panel is the first 4-new-born leaves of the tomato plants. **(C)** Less TYLCV accumulation in the MU1 inoculated susceptible plants but not in the resistant plants. Total TYLCV copies were measured by qPCR analysis at 15 dpi. Error bars represented SE of three biological replicates and significant differences by Student’s *t* test (*, p<0.05). **(D)** *SlLNR1* was down regulated by the TYLCV infection but increased by the MU1 in the susceptible plants. The RNA sample was extracted at 15 days after inoculated by the infectious clone. The relative expression of *SlLNR1* was measured by qRT-PCR. The tomato *actin* gene was set as reference gene. Error bars represented SE of three biological replicates. Duncan’s multiple range test was conducted, and the different letters in graphs indicate significant differences between treatments (p<0.05).

Furthermore, we generated mutated TYLCV infectious clones to evaluate the importance of the 25-nt sequence complementarity between the IR and *SlLNR1* in TYLCV-caused disease symptoms. Since the IR is also involved in replication and activation of gene expression, we generated 9 mutants that may disrupt complementarity between the IR and *SlLNR1* (S6A Fig). Among them, a mutant (named as MU1) exhibits similar activities of replication (TYLCV *AC1* gene expression) and promoter (*GUS* reporter gene expression) in *N*. *benthamiana* (S6B and S6C Fig), in which 2 nucleotides within the 25-nt IR region were modified (Fig 4A). The tomato plants inoculated by the wild isolate appeared yellow, curled and shrinking, while those with MU1 has only slight pathogenic phenotypes which is comparable to the uninfected control (Fig 4B). The virus accumulation in the susceptible plant treated with MU1 was lower than those with wild isolates, but not in the resistant plant (Fig 4C). Then we examined the expression of *SlLNR1* in the infected tomato plants, which was dropped significantly in the susceptible plants treated by the wild isolate but increased in those with MU1. However, expression of *SlLNR1* in the resistant plant was induced by inoculation with both of the WT and MU1 (Fig 4D). As the IR region serves as the promoter for the bi-directional promoter, the transcriptional activity of the virus genes was analyzed in *N*. *benthamiana*. The transcripts of *AV2* and *AC1* were analyzed by qRT-PCR on the samples of *N*. *benthamiana* agro-inoculated with the MU1 and wild type isolate. The transcripts amount of *AV2* and *AC1* were not changed in the MU1 compared with the wild type (S6D Fig). These results together validated that the vsRNAs from the 25-nt IR segment targeted the *SlLNR1*.

To examine vsRNA-mediated cleavage of *SlLNR1*, we performed a 5’-RACE analysis on mRNA isolated from the TYLCV-infected susceptible tomato plants and detected a dominant *SlLNR1*-derived product. Sequencing showed the cleavage may occur at the 9^th^ nucleotide from the 5’ end of the highly abundant siRNA (-2752-21) (Fig 2A). It has been reported that siRNA-guided RNA cleavage usually occurs between nucleotides 10^th^ and 11^th^ from the 5’ end of this siRNA [43]. Thus, we speculate that other less abundant vsRNAs, e.g. siRNA (-2753-21), may participate in the cleavage of *SlLNR1* or siRNA (-2752-21)-mediated cleavage may undergo a distinct manner.

### *SlLNR1* contributes to the normal development and TYLCV resistance in tomato

**Fig 5:**
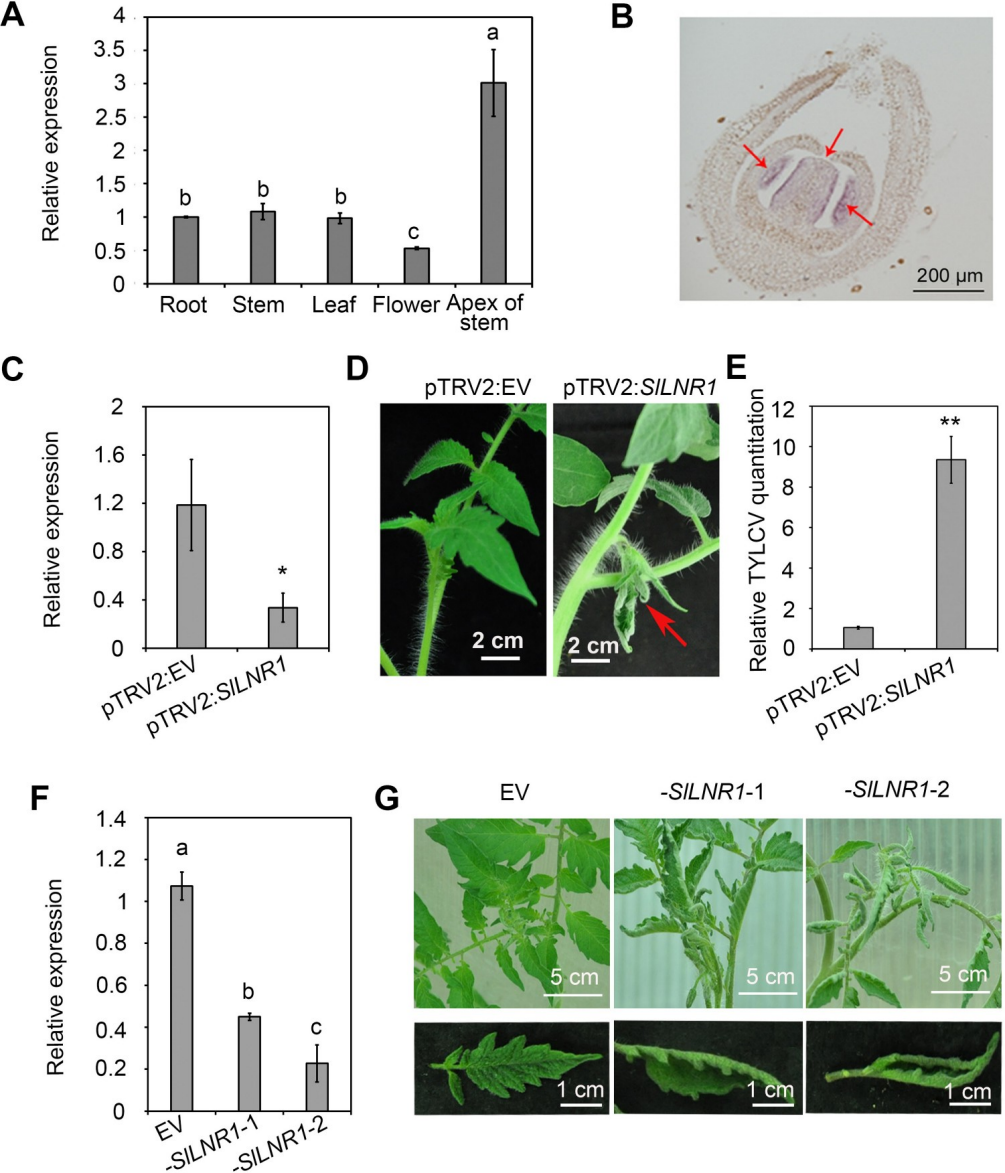
The *SlLNR1* is important for tomato normal leaf development and resistance to TYLCV. **(A)** qRT-PCR analysis of *SlLNR1* expression in different tissues. Total RNA was isolated from the resistant tomato cultivars. Error bars represented SE of three biological replicates. Different letters on the bars designate statistically significant differences (P<0.05) according to Duncan’s multiple range test. **(B)** *In situ* hybridization of the *SlLNR1* RNA. An 896-bp *SlLNR1* fragment was used as the probe for *in situ* hybridization of *SlLNR1* RNA (Bar = 200 μm). **(C)** The *SlLNR1* was silenced in the resistant tomato. The relative expression of *SlLNR1* was measured by qRT-PCR and calculated in relation to the EV inoculated samples at 15 dpi according to the ^ΔΔ^Ct method. The tomato *actin* gene was set as reference gene. Error bars represented SE of three biological replicates and significant differences by Student’s *t* test (*, p<0.05; **, p<0.01). **(D)** Abnormal new-born leaves in the *SlLNR1-*silenced tomato plants. The photos were taken at 15 days after agroinfiltration of pTRV2:*SlLNR1* on the tomato resistant cultivar. The arrow indicates the curling leaves. pTRV2 was used as a negative control and pTRV2:*SlLNR1* indicates the silenced plants. (**E**) More TYLCV accumulation in the pTRV2:*SlLNR1* plants. Total relative TYLCV genomic DNA amounts in pTRV2:*SlLNR1* and EV inoculated plants were measured by qPCR analysis and calculated in relation to the EV inoculated samples according to the ΔΔCt method. The tomato actin gene was set as reference gene. Error bars represented SE of three biological replicates and significant differences by Student’s t test (*, p<0.05; **, p<0.01). **(F)** The expression of *SlLNR1* was downregulated in the RNAi plants. *SlLNR1* relative expressional levels were measured by qRT-PCR and and calculated in relation to the EV-transgenic plants according to the ^ΔΔ^Ct method. The tomato *actin* gene was set as reference gene. Different letters on the bars designate statistically significant differences (P<0.05) according to Duncan’s multiple range test. **(G)** Abnormal phenotypes in the *SlLNR1*-silenced plants. The photos were taken at 2 months after germination. The *-SlLNR-1/2* are *SlLNR1* RNAi plants and EV indicate pCAMBIA2301 transgenic plants.

To examine if this lncRNA had a potential biological function, we analyzed its accumulation in different plant tissues, which showed that it accumulated at the highest level in the apex of stem (Fig 5A). This tissue specific expression patterns were further confirmed by *in situ* RNA hybridization (Fig 5B). To further explore the biological role of *SlLNR1*, we knocked down the expression of the gene using TRV-based virus-induced gene silencing in the TYLCV-resistant cultivar CLN2777A [44]. The repression of *SlLNR1* by pTRV2:*SlLNR1* was confirmed by qRT-PCR analysis showing approximately 50% downregulation compared to pTRV2:EV-infiltrated plants (Fig 5C). *SlLNR1*-silenced tomato seedlings developed curled and stunted new leaves about 15 dpi (Fig 5D). The TYLCV-resistant levels were also severely impaired in the *SlLNR1*-silenced tomato seedlings (Fig 5E). Furthermore, stable transgenic *SlLNR1*-silenced tomato lines were generated by expression of a selected fragment in a susceptible cultivar. Two independent transgenic lines were identified, in which *SlLNR1* expression was decreased about 49%-77% (Fig 5F). Both of them showed abnormal phenotypes including the inward rolling up leaves and curl of blade edge. Additionally, the phenotype abnormality was seemed to correlate with the degree of repression of the *SlLNR1* (Fig 5G).

**Fig 6:**
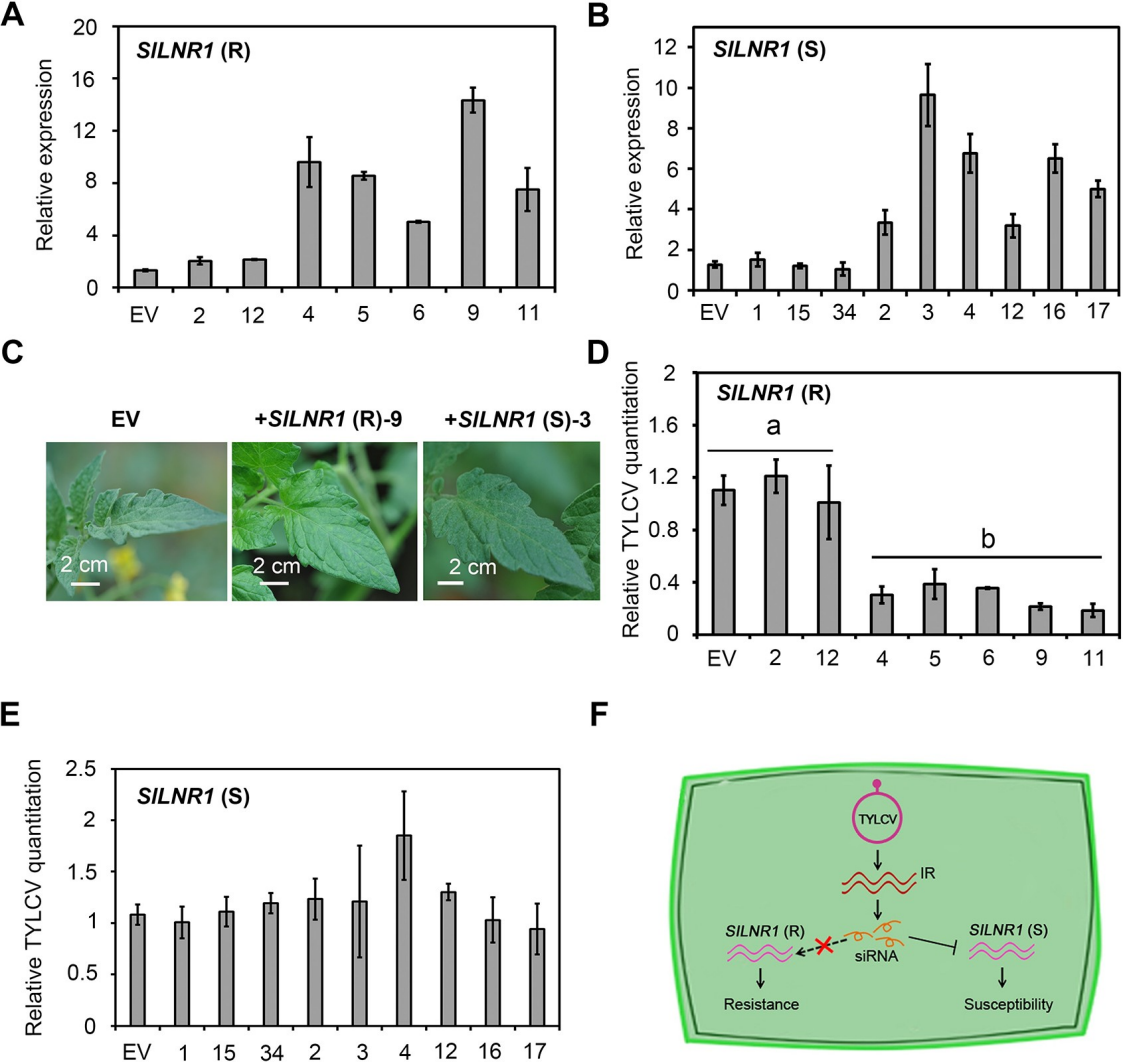
*SlLNR1(R)*, but not *SlLNR1(S)*, contributes the resistance to TYLCV. **(A)** Various expressional levels of *SlLNR1(R)* in the transformed plant*s*. *SlLNR1(R)* relative expressional levels of the T3 generation were measured by qRT-PCR and and calculated in relation to the EV-transgenic plants according to the ^ΔΔ^Ct method. The tomato *actin* gene was set as reference gene. In the 9 analyzed lines, -2 and -12 show similar levels to the EV-transgenic plants (pCAMBIA2301). -4, -5, -6, -9, and -11 are five independent overexpression lines. **(B)** Various expressional levels of *SlLNR1(S)* in the transformed plant*s*. *SlLNR1(S)* relative expressional levels of the T3 generation were measured by qRT-PCR and and calculated in relation to the EV-transgenic plants according to the ^ΔΔ^Ct method. The tomato *actin* gene was set as reference gene. **(C)** Leave phenotypes of *SlLNR1*-transgenic tomato plants. Tomato plants (S) were transformed with the EV pCAMBIA2301 (EV, as a negative control), the sense *SlLNR1(R)* or *SlLNR1(S)* construct. Both *SlLNR1* overexpression (+*SlLNR1(R)*-9, +*SlLNR1(S)*-3 as an example) were used for observations. The photos were taken at 2 months after germination. **(D)** TYLCV accumulation levels in the *SlLNR1(R)* overexpressed plants. Total relative TYLCV genomic DNA levels in the transformed plants inoculated with TYLCV infectious clone (15 dpi) was measured by qPCR analysis and calculated in relation to the EV inoculated samples according to the ^ΔΔ^Ct method. The tomato *actin* gene was set as reference gene. Different letters on the bars designate statistically significant differences (P<0.05) according to Duncan’s multiple range test. **(E)** TYLCV accumulation levels in the *SlLNR1(S)* overexpressed plants. Total relative TYLCV genomic DNA levels in the transformed plants inoculated with TYLCV infectious clone (15 dpi) was measured by qPCR analysis and calculated in relation to the EV inoculated samples according to the ^ΔΔ^Ct method. The tomato *actin* gene was set as reference gene. **(F)** Tentative model for interactions of TYLCV siRNAs and tomato *SlLNR1*. TYLCV produces a set of siRNAs that are derived from its IR. The IR and siRNAs are virulent and may cause abnormal symptoms on plants. The siRNAs target tomato *SlLNR1*, a lncRNA that is critical to virus resistance and normal development of leaves, to suppress its expressional levels in susceptible plants. In contrast, the *SlLNR1* loses siRNAs-targeted sites in the resistant cultivar.

To verify the TYLCV resistant function of *SlLNR1*, we generated the transgenic tomato lines overexpressing *SlLNR1(R)* or *SlLNR1(S)*. In total, we obtained 5 independent transgenic lines for *SlLNR1(R)* (Fig 6A) and 6 for *SlLNR1(S)* (Fig 6B), respectively. All the overexpressed lines exhibited normal phenotypes (Fig 6C). Then, we assessed TYLCV accumulation in these transgenic tomato plants. Viral DNA accumulation was significantly reduced in the plants with *SlLNR1(R)*-overexpressed lines compared to the EV trasngenic plants (Fig 6D). However, the amounts of virus were not repressed in all the *SlLNR1(S)*-transformed plants (Fig 6E). It was supposed only the *SlLNR1(R)* escaping the targeting of vsRNAs has the TYLCV resistant function. Taken together, these results suggest that *SlLNR1* is not only required for normal plant development, but also negatively regulates the accumulation of TYLCV, both of which may contribute to disease development in tomato (Fig 6F).

## Discussion

A zigzag model has been widely accepted to encompass the complicated arms race between host plants and bacterial/fungal pathogens [45]. In plant-virus interactions, RNA silencing pathway senses viral RNAs using Dicer-like enzyme to produce vsRNAs and restrict virus replication, which is reminiscent of pattern-triggered immunity (PTI) in the zigzag model [15–18]. Viruses produce RNA silencing suppressors to counter this host defense mechanism. This study provides another example to illustrate that TYLCV may generate vsRNAs to interfere with host gene expression to modulate symptoms and achieve high-level infection. To cope with the virus infection, the resistant plant has developed an adaptive genomic change to escape targeting by vsRNAs. Thus, RNA silencing-mediated plant-virus interaction may play an important role in the evolution of both the viral and host genomes.

We performed sequence alignment between the TYLCV-derived vsRNAs and tomato transcripts to identify the pathogenic determinant vsRNAs. Consistent with the previous reports [36–38], our deep sequencing analysis show that the vsRNAs were unequally distributed throughout the viral genome with many hotspots and relatively low number was originated from the IR. Interestingly, among them, a 25-nt IR segment (nt 2730–2754) shows near-perfect complementarity sequence with tomato *SlLNR1* gene. During the validation of its virulent functions, we found that expressing of the IR, this segment alone or the siRNA in tomato caused similar phenotypes to the whole virus while mutants of the complementary sequences abolish the virulence. Deep sequencing results revealed that many 21–24 nt vsRNAs derived from this segment, suggesting that it is a vsRNAs-generating hotspot. Although we still could not conclude which particular siRNA plays dominant roles or they act in synergic manner, a high abundance 21-nt antisense siRNA (-2752-21) was selected as example for analysis. Indeed, it was detectable in the nature field diseased plants, TYLCV-infected samples or the IR/25-nt-segment-expressing plants. Its expressional levels were highly induced during infection and negatively related to expression of *SlLNR1*. Thus, we speculate that the vsRNAs derived from this segment may target and regulate host gene expression to trigger susceptibility, leading to viral symptoms.

vsRNAs-triggered susceptibility has been observed in several cases in RNA viruses and viroids. Peach latent mosaic viroid (PLMVd) and Potato spindle tuber viroid (PSTVd) compose of only a short non-protein-coding RNA genome and cause visible symptoms during infection. A PSTVd-sRNA that is essential for virulence targets tomato two *callose synthase* genes [20] and two PLMVd-sRNAs may cleavage the mRNA encoding the chloroplastic heat-shock protein 90 [46]. CMV deploys a 22 nt Y-sat-derived siRNA to silence host *ChlI* gene to cause yellowing symptoms [19, 20]. Like CMV and these two viroids, TYLCV as a DNA virus is also an inducer and target of host RNA silencing. Here we showed that its IR is bidirectionally transcribed, suggesting that the region may form a dsRNA precursor of primary vsRNAs. In another DNA geminivirus Cabbage leaf curl virus, the vsRNAs biogenesis is likely independent of RNA-dependent RNA-polymerase (RDR) in *Arabidopsis* and the primary vsRNAs may trigger RDR-dependent generation of secondary siRNAs [38]. Interestingly, the known TYLCV resistance *Ty-1* and *Ty-3* genes encode for RDRs, which are similar to *Arabidopsis* RDR3, -4, and -5 and responsive for amplification of the siRNA signal. Tomato lines carrying *Ty-1/3* genes confer resistance through enhanced gene silencing, and then exhibit high levels of TYLCV-derived siRNAs [27]. Thus, we speculate that TYLCV may reinvent the generated siRNAs as virulent factors to counter the host resistant pathway. The biogenesis of these pathogenic determinant vsRNAs and its interactions with host immune systems remain to be analyzed.

Over recent decades, pathogen effector proteins (effectors) have received much attention because of their critical roles in plant-microbe interactions and the zigzag model. The effectors interfere with host defense pathways by directly interacting with host immunity components to benefit pathogen infection, which is called effector-triggered susceptibility (ETS) [45]. Analogy to these, siRNAs produced by different pathogens may translocate into the host cells to induce silencing of host immunity genes. Thus, the term of ‘siRNA effecttor’ was proposed [47, 48] and siRNA effectors have been reported in several pathosystems, including interactions of *B*. *cinerea* and hosts tomato/*Arabidopsis* [21], nematode parasites and mammalian cells [49], *Escherichia coli* and *Caenorhabditis elegans* [50], and *Wolbachia* and host insects [51]. The study here provides many lines of evidence to show that siRNA effectors are also shared by TYLCV, a kind of plant DNA virus, suggesting of the wide spread of siRNA effectors and a common pathogenic strategy in diverse parasites. Future identification of similar siRNAs in different pathogens will help identify host immunity genes and unravel molecular mechanisms of plant-parasite interactions.

This IR segment (nt 2730–2754) has only one mismatch with tomato *SlLNR1* gene in a susceptible cultivar and is a vsRNAs-generating hotspot. Interestingly, tomato *SlLNR1* allele gene in a resistant cultivar loses a 14-nt fragment of this complementarity region, indicative a possible escaping mechanism during molecular arms race. Expression of *SlLNR1* gene was significantly downregulated during infection in the resistant cultivar, but upregulated in the susceptible cultivar. Its expression was silenced in plant expressing the IR or the 25-nt-segment and its cleavage site was also determined. By bioinformatical analysis, one EST (GenBank ID: BF097137.1) was another potential target of the segment-derived vsRNAs with three mismatch, which was also a lncRNA. However, we noticed that its expression was not altered by in the above experiments and the gene is unique in tomato based on sequence similarity searching in other plants. At the same time, genetic evidence of function loss and gain show that *SlLNR1* gene was critical for leave normal development and TYLCV resistance. We found that it has both sense and antisense transcripts, among which the antisense form was complementary to the 25-nt segment. Plant lncRNAs have been implicated in a wide of biological functions through various mechanisms, including acting as precursors and sponges of siRNA or directing histone modification of transcripts [52]. *SlLNR1* gene does not belong to the six known *Ty* loci. It is likely involved in different networks related to TYLCV resistance. Its precise role and crosstalk with the *Ty* genes are now essential.

It has been reported that many genes are involved in TYLCV resistance and RNA silencing is a major resistant strategy. Among the six known *Ty* loci, *Ty-1* and *Ty-3* encode RDRs which intensify RNA silencing pathway to boost disease resistance [25, 27]. TYLCV have RNA silencing suppressors to counter the defense. Here, we show that interaction between TYLCV-derived vsRNAs and host lncRNA mediates disease symptoms and severity, indicative of a novel evolutionary arms race between the vsRNAs and *SlLNR1* gene. It is noteworthy to address whether and how *Ty* genes and other defense regulators are involved through population genetics.

TYLCV represents a group of DNA geminivirus that can cause severe damages and yield loss to many important crops [1, 2]. The lack of understanding of the viral disease or the host resistance mechanisms has hampered the development of effective approaches to control the viral diseases. The DNA nature of the viral genome also makes this group of viruses difficult to eliminate using transgenic RNA silencing technologies. This study provides a plausible, vsRNA-lncRNA based disease mechanism that should open new avenues in developing new viral control strategies. For instance, viral disease resistance could potentially be achieved by silencing the expression of the specific vsRNAs, introducing a silencing-resistant version of the target lncRNA or genetic editing the lncRNA. It could also help to develop molecular markers for selecting TYLCV-resistant plant materials during breeding programs.

## Materials and methods

### Plant, microbial strains, virus and infection assays

The TYLCV-resistant tomato cultivar CLN2777A (*Ty-2/Ty-2*) and susceptible cultivar JS-CT-9210 were grown in a growth chamber under 23/19°C (day/night) and 16/8 h (light/dark) conditions [53]. Tobacco (*Nicotiana tabacum*) cultivar K326 and *N*. *benthamiana* were grown in a 25/22°C (day/night) while cotton plants (*Gossypium hirsutum* cv. Junmian 1) were grown in a 27/25°C growth chamber under a 12 h photoperiod. Transgenic plants were grown in the same conditions with the recipient plants.

*A*. *tumefaciens* strains GV3101 were grown at 28°C in LB supplemented with 50 μg/mL kanamycin and 50 μg/mL rifampicin. The wild TYLCV infectious clone TYLCV (CN:SH2) and mutated clones were used to inoculate tomato plants with agro-inoculated method [37]. The phenotype was observed at 15 days post inoculation (dpi) and the accumulation of TYLCV was verified by qPCR, and at least 50 plants were inoculated for each infection clone.

Whiteflies viruliferous of the TYLCV-IL strain were propagated and maintained with tomato plants in insect-proof greenhouse. Three- to four-true leaf stage tomato plants were inoculated with viruliferous whiteflies in insect-proof cages for 3 days, which were then treated with an insecticidal imidacloprid to kill the whiteflies. The phenotype was observed at 15 dpi and the accumulation of TYLCV was verified by qPCR.

### Small RNA library construction for sequencing and data analysis

Tomato plants inoculated by TYLCV infectious clone at 15 dpi and pTRV2:IR and EV pTRV2 at 20 dpi were collected in liquid nitrogen. Total RNA was extracted using the RNA simple purification Kit (Tiangen, China) and used for library preparation by means of the TruSeq Small RNA library preparation kit from Illumina. Then sRNAs were sequentially ligated to a 3’ adapter and a 5’ adapter. After each ligation step, sRNAs were purified using 15% denaturing PAGE. The final purified ligation products were reverse transcribed into cDNA using Superscript III reverse transcriptase (Invitrogen). The first strand DNA was PCR amplified using Taq polymerase (Takara, Japan) and DNA amplicons from each library were purified and separately submitted for high-throughput sequencing using the Hi-seq 2000 platform (Illumina, San Diego, CA).

The raw sequencing data were firstly filtered out the adapter sequences by in-house Perl scripts. Then, the filtered reads with 18–25 nt in length were aligned with TYLCV genome sequences (GenBank ID: AM282874), TRV1(GenBank ID: AF406990) or TRV2 (GenBank ID: AF406991) and *SlLNR1* by bowtie software [54]. Only reads with perfect match were selected for further analysis.

### Northern blot analysis

The RNA-blot analyses for detecting the siRNA were performed as described previously [55]. Total RNA was extracted using TRIzol reagent (Invitrogen™) following the manufacturer's instructions. The concentration of all the samples should be adjusted to 3μg/μL and the total loading quantity of RNA was 60 μg. The RNA was resolved on a 14% denaturing 8 M urea-PAGE gel and then transferred and chemically crosslinked onto a Hybond N+ membrane using N-(3-Dimethylaminopropyl)-N'-ethylcarbodiimide hydrochloride. The probe for siRNA detection was 5'-AATTCAAAATTCAAAAATCAA-3', the 5’ and 3’ end was labeled with biotin and the LNA modified at the 7^th^, 12^nd^ and 14^th^ site. The *U6* probe is 5'-AGGGGCCATGCTAATCTTCTC-3' with the biotin labeled at the 5’ and 3’ end. The Northern blot was carried at 50°C for 16h. The detection of immobilized nucleic acids was carried with the Chemiluminescent Nucleic Acid Detection Module Kit (Thermo, cat.89880).

### Vector construction

All the used oligonucleotides and generated constructs were listed and described in S1 and S2 Tables, respectively. There is a *Sac* I digestion site in the genome sequence of TYLCV. To construct the mutant infectious clones of TYLCV, the full length of TYLCV was amplified with the primers introducing the mutant sites in the IR region and *EcoR* I digestion sites, which then was digested by the *EcoR* I and *Sac* I to obtain the 2185 bp fragment and inserted to the pCAMBIA2301 vector. The full length of TYLCV was digested by the *EcoR* I and the 2781 bp enzyme-digested products were constructed on the first constructed vector, and the position of the fragment should be confirmed.

### Real-time PCR

For the IR, *AV2*, *AC1* and *SlLNR1* expression analysis, the RNA of young leaves or other organs were extracted with PLANT simple RNA extraction kit (TIANGEN) and was reverse transcripted by the HiScript II Q RT SuperMix for qPCR (Vazyme). For the transient expression analysis, total RNA was extracted from the injection site of leaves at 48 hours after agro-infiltration. The extracted total RNA samples were treated with DNase to avoid the potential DNA contamination.

For the VIGS analysis, the new emerging leaves from the pTRV2:*SlLNR1* plants at 15 dpi were used to extract RNA, which was subsequently used to determine the expression level of target gene by qRT-PCR. The VIGS-treated plants were inoculated with TYLCV infection clone, and the DNA was extracted at 15 days after infected for determining the TYLCV accumulation by qPCR. PCR thermal cycler qTOWER 2.0/2.2 (Analytik Jena, Germany) was used for qRT-PCR and qPCR analysis with PCR conditions consisting of denaturation for 10 s at 95°C, annealing for 15 s at 60°C, and extension for 20 s at 72°C for 35 cycles. The expression levels of selected genes were normalized to tomato *actin* gene (AB199316) expression. All qRT-PCR expression assays were independently performed and analyzed three times under identical conditions.

### RT-PCR and RACE analysis

The sense or antisense transcripts of IR were confirmed by RT-PCR with the cDNA reverse-transcribed with the IR specific primers and the RNA template was also amplified with the same primers to validate no DNA contamination. The 5’ flanking region of the sense transcripts of *SlLNR1* were obtained by RNA ligase-mediated rapid amplification of 5' cDNA ends First Choice ^®^ RLM-RACE Kit (Invitrogen, USA), according to the instructions of the manufacturer, and the 3’ end was verified by 3’ RACE PCR kit (TAKARA). Full sense transcript of the *SlLNR1* was amplified with the primers designed according to the joint sequence by RT-PCR. The antisense transcripts of *SlLNR1* were detected by strand-specific RT-PCR with the cDNA reverse-transcribed with the specific sense primers [56]. All the above PCR products were cloned into pMD19-T (Takara) and validated by sequencing.

To verify the cleavage site of *SlLNR1*(-) by siRNA, total RNA was isolated from the new born leaves with TYLCV infection clone inoculated plants for 36 days and the control plants. And the 5’-RACE was performed using FirstChoice ^®^ RLM-RACE Kit (Invitrogen, USA), according to the instructions of the manufacturer. The RNA was reversed transcribed using random primers and then the 5’-end of cDNA was amplified using the 5’ outer primer and the *SlLNR1*(-) specific reverse primer for the first round PCR. The amplified product was used for subsequent nested PCR with the inner primer and the *SlLNR1*(-) specific nested reverse primer. The PCR products were detected by agarose gel electrophoresis. The target fragments were cloned into pGEM-T Easy vectors (Promega, USA) and sequenced.

### *In situ* hybridization

The specific cDNA fragment of *SlLNR1* was amplified and inserted the into pGEM-T Easy (Promega) for sequencing. The probe was then generated by primers T7-F and R, which then was transcribed in vitro from the T7 promoter with T7 RNA polymerases using the digoxigenin RNA-labeling kit (Roche). Tissues for *in situ* hybridization was fixed overnight in 4% (wt/vol) paraformaldehyde in phosphate buffer, pH 7.0, and embedded in Paraplast Plus (Sigma). Nonradioactive RNA in situ hybridization with digoxigenin-labeled sense and antisense probes was performed on 8-mm sections of different root parts as described [57].

### Tobacco rattle virus (TRV)-based expression system for gene silencing and overexpression

*SlLNR1* was silenced by tobacco rattle virus (TRV)-based gene silencing system. For agroinfiltration, an equal volume of *Agrobacteria* containing of pTRV1 or pTRV2:*SlLNR1* was mixed and infiltrated into the cotyledons of tomato seedlings at the cotyledon stage with 1 mL syringe. The agroinfiltration of pTRV1 with pTRV2:PDS and pTRV1 with empty pTRV2 served as positive control and negative control respectively. The TRV based expression system was also employed to mediate the overexpression of IR and 4×25-nt-fragment (4TR) in tomato plants. The plants were from 15-days after infiltration and the samples were collected for RNA extraction.

### *Agrobacterium*-mediated transient expression in *N*. *benthamiana*

For transient expression assays, *Agrobacterium* cells grown overnight were harvested and resuspended in infiltration media (10 mM MgCl_2_, 10 mM MES, 200 mM acetosyringone) to an O.D. value of 1.0 and cultured at room temperature for 4 h. Then the suspensions were infiltrated into the leaves of *N*. *benthamiana* using a needleless syringe. The samples were collected for RNA extraction or GUS activity analysis at 48 hours after agroinfiltration.

### *Agrobacterium*-mediated tomato and tobacco transformation

*Agrobacterium*-mediated tomato and tobacco transformation was performed in accordance to the protocol with some modifications [57, 58]. And the susceptible tomato line AC and tobacco cultivar K326 was used for the transformation work of construct for overexpression.

## Supporting information

S1 Fig The expressional levels of the IR and the viral accumulation of TRV in the infected plants. (DOCX)

S2 Fig Distribution of siRNAs and phenotypes induced by the IR in tobacco and cotton. (DOCX)

S3 Fig Determination of *SlLNR1*. (DOCX)

S4 Fig Alignment of *SlLNR1* in two tomato cultivars. (DOCX)

S5 Fig *SlLNR1* associated siRNAs derived from the tomato plants inoculated by pTRV:IR and EV. (DOCX)

S6 Fig The replication and promoter activities of different TYLCV mutants in *N*. *benthamiana*. (DOCX)

S1 Table Oligonucleotides used in the study. (DOCX)

S2 Table The constructs used. (DOCX)
