# Supplementary material for: Tomato yellow leaf curl virus intergenic siRNAs target a host long noncoding RNA to modulate disease symptoms
Source: PLoS Pathog. 2019 Jan 22;15(1):e1007534. doi: 10.1371/journal.ppat.1007534 (PMC6366713; doi:10.1371/journal.ppat.1007534)
Supplement: S1 Fig — (DOCX) [file ppat.1007534.s001.docx]

Supporting Information





S1 Fig. The Expressional levels of the IR and the viral accumulation of TRV in the infected plants. (A) Expressional levels of the IR. qRT-PCR analysis of the IR transcripts in tomato plants inoculated with EV pTRV2 (EV) and pTRV2:IR (IR) at 15 dpi. The transcript levels of selected gene were normalized to tomato *actin* gene. S and R mean the TYLCV-susceptible and -resistant line respectively. Error bars represented SE of three biological replicates. (B) The relative TRV quantitation during infection by transcriptome sequencing. The TRV amounts were calculated by ration of TRV-derived siRNAs (including TRV1 and TRV2 genomes), and total siRNAs number determined by small RNA sequencing. Error bars represented SE of two biological replicates. (C) The relative TRV quantitation during infection by qRT-PCR. The transcripts of the genes encoding TRV1 replicase and TRV2 2b in the inoculated plants were used to evaluate the TRV quantitation by qRT-PCR. Error bars represented SE of three biological replicates.
