# Supplementary material for: Tomato yellow leaf curl virus intergenic siRNAs target a host long noncoding RNA to modulate disease symptoms
Source: PLoS Pathog. 2019 Jan 22;15(1):e1007534. doi: 10.1371/journal.ppat.1007534 (PMC6366713; doi:10.1371/journal.ppat.1007534)
Supplement: S2 Fig — (DOCX) [file ppat.1007534.s002.docx]

Supporting Information


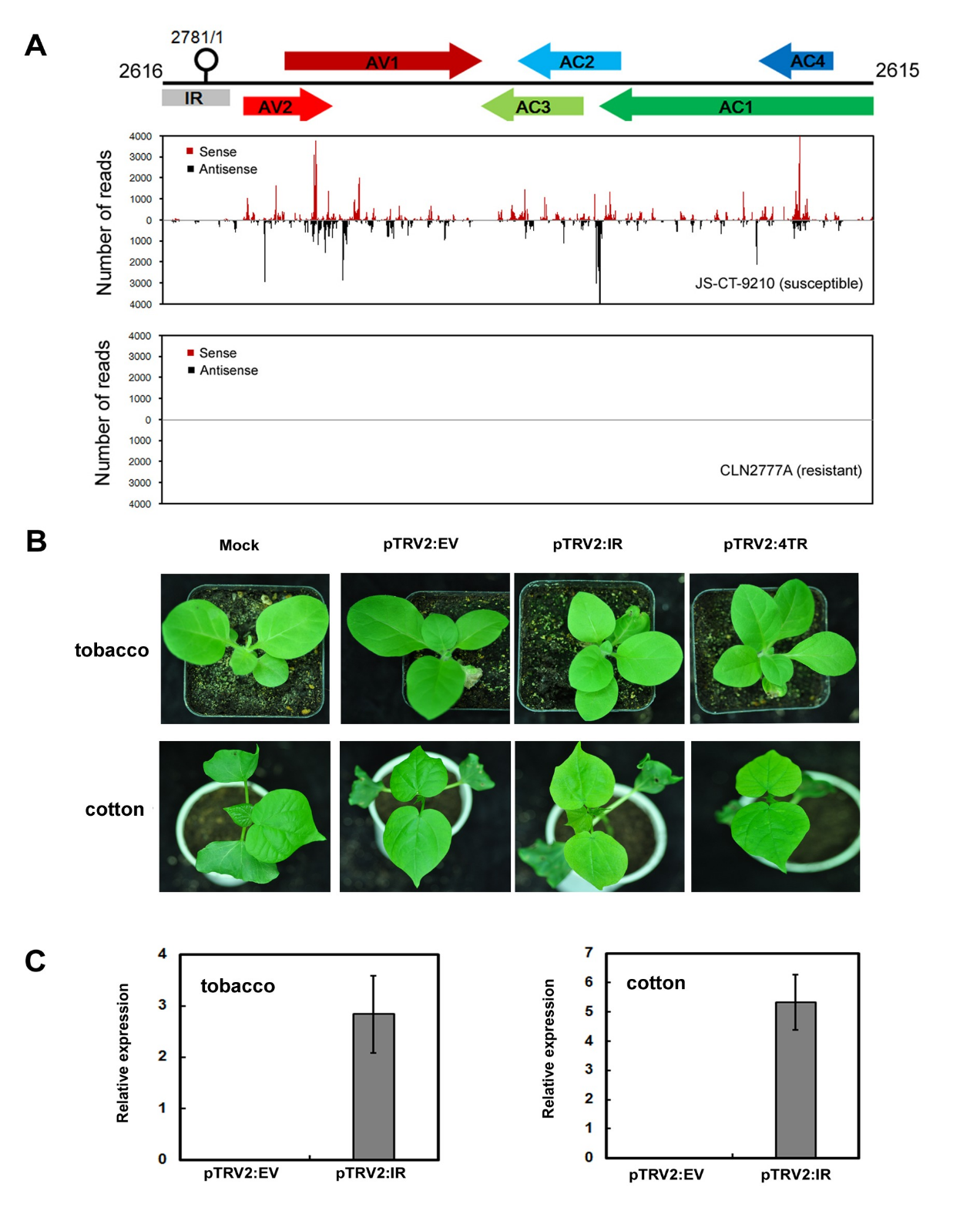


S2 Fig. Distribution of siRNAs and phenotypes induced by the IR in tobacco and cotton. (A) SiRNAs generated from TYLCV during infection. Location and frequency of TYCLV-derived small RNAs were mapped to the genome in either sense (above the x-axis) or anti-sense (below the x-axis) orientation. (B) Phenotypes of tobacco and cotton plants inoculated with pTRV2:EV, pTRV2:IR and pTRV2:4TR. The photos were taken at 15 dpi. (C) The transcriptional levels of the IR. qRT-PCR analysis of the IR transcriptional levels in tobacco and cotton plants inoculated with pTRV2:IR at 15 dpi. Cotton *UBQ14* and tobacco *EF1a* genes were set as internal control.
