# Supplementary material for: Tomato yellow leaf curl virus intergenic siRNAs target a host long noncoding RNA to modulate disease symptoms
Source: PLoS Pathog. 2019 Jan 22;15(1):e1007534. doi: 10.1371/journal.ppat.1007534 (PMC6366713; doi:10.1371/journal.ppat.1007534)
Supplement: S3 Fig — (DOCX) [file ppat.1007534.s003.docx]

Supporting Information





## **S3 Fig. Determination of *SlLNR1*.** **(A)** Sequence determination of *SlLNR1* with 5’ RLM RACE PCR. The 5’ *SlLNR1* region was obtained by 2-rounds RLM-RACE (F, first; S, Second) and validated by sequencing of PCR products. **(B)** The 3’ region generated by 3’ RACE PCR.
