# Supplementary material for: Tomato yellow leaf curl virus intergenic siRNAs target a host long noncoding RNA to modulate disease symptoms
Source: PLoS Pathog. 2019 Jan 22;15(1):e1007534. doi: 10.1371/journal.ppat.1007534 (PMC6366713; doi:10.1371/journal.ppat.1007534)
Supplement: S4 Fig — (DOCX) [file ppat.1007534.s004.docx]

Supporting Information


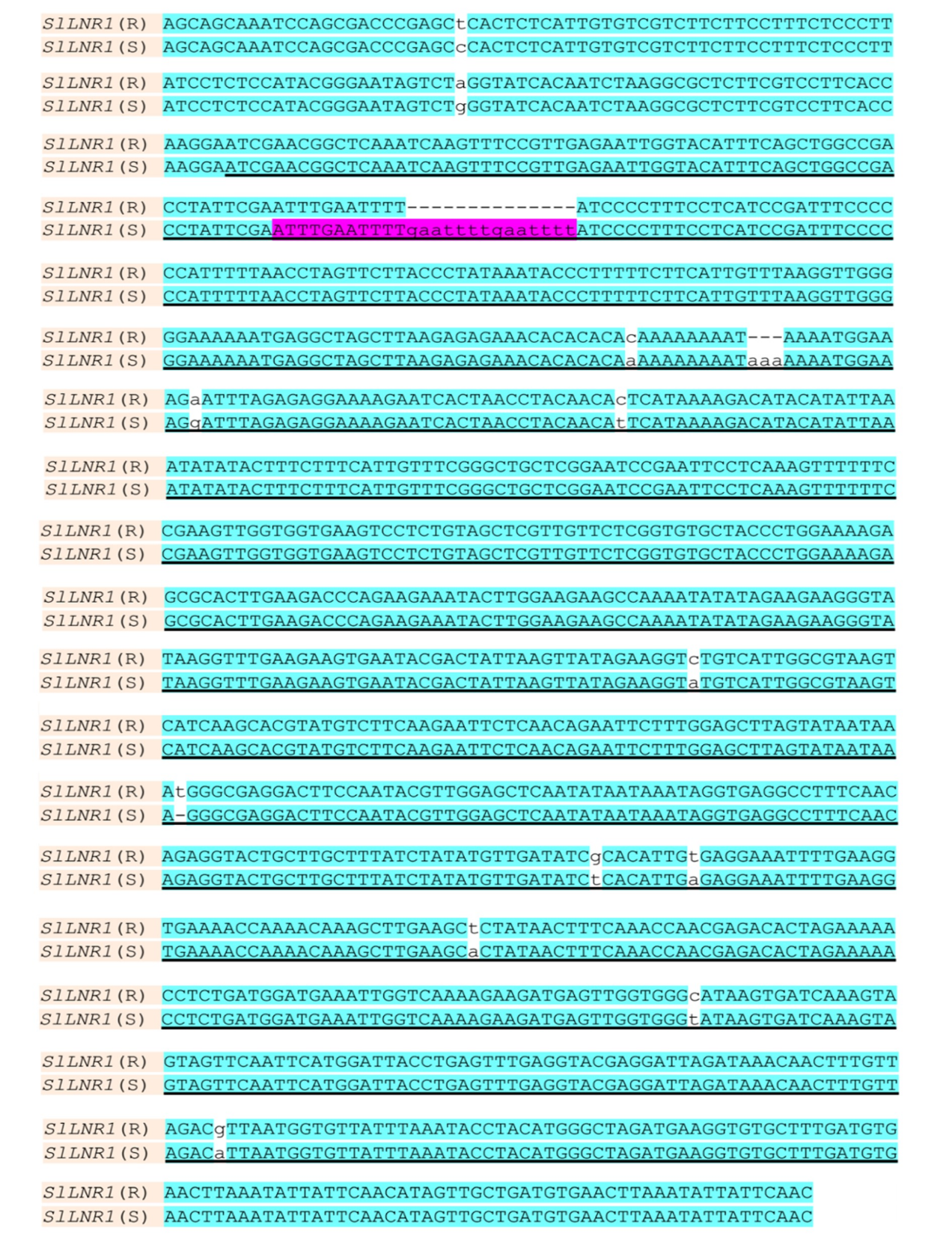


S4 Fig. Alignment of *SlLNR1* in two tomato cultivars. *SlLNR1* from CLN2777A (R) and JS-CT-9210 (S) were shown. The antisense-transcript of *SlLNR1* in susceptible cultivar was underlined. The 25-nt-fragment, which was corresponding to TYLCV nt 2732-2752, was purple labeled and a 14-nt deletion in resistant cultivar was indicated.
