# Supplementary material for: Tomato yellow leaf curl virus intergenic siRNAs target a host long noncoding RNA to modulate disease symptoms
Source: PLoS Pathog. 2019 Jan 22;15(1):e1007534. doi: 10.1371/journal.ppat.1007534 (PMC6366713; doi:10.1371/journal.ppat.1007534)
Supplement: S5 Fig — (DOCX) [file ppat.1007534.s005.docx]

Supporting Information


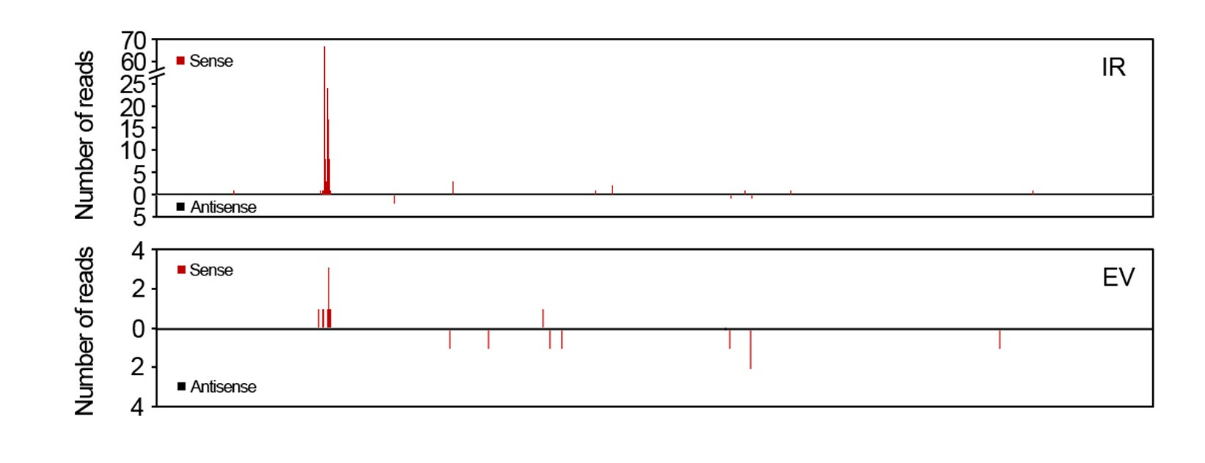


S5 Fig. *SlLNR1* associated siRNAs derived from the tomato plants inoculated by pTRV:IR and EV. The data derived from small RNA sequencing of pTRV2 (EV) or pTRV2:IR (IR) inoculated plants was aligned to *SlLNR1* and the number indicates the aligned siRNA reads.
