# Supplementary material for: Tomato yellow leaf curl virus intergenic siRNAs target a host long noncoding RNA to modulate disease symptoms
Source: PLoS Pathog. 2019 Jan 22;15(1):e1007534. doi: 10.1371/journal.ppat.1007534 (PMC6366713; doi:10.1371/journal.ppat.1007534)
Supplement: S6 Fig — (DOCX) [file ppat.1007534.s006.docx]

Supporting Information


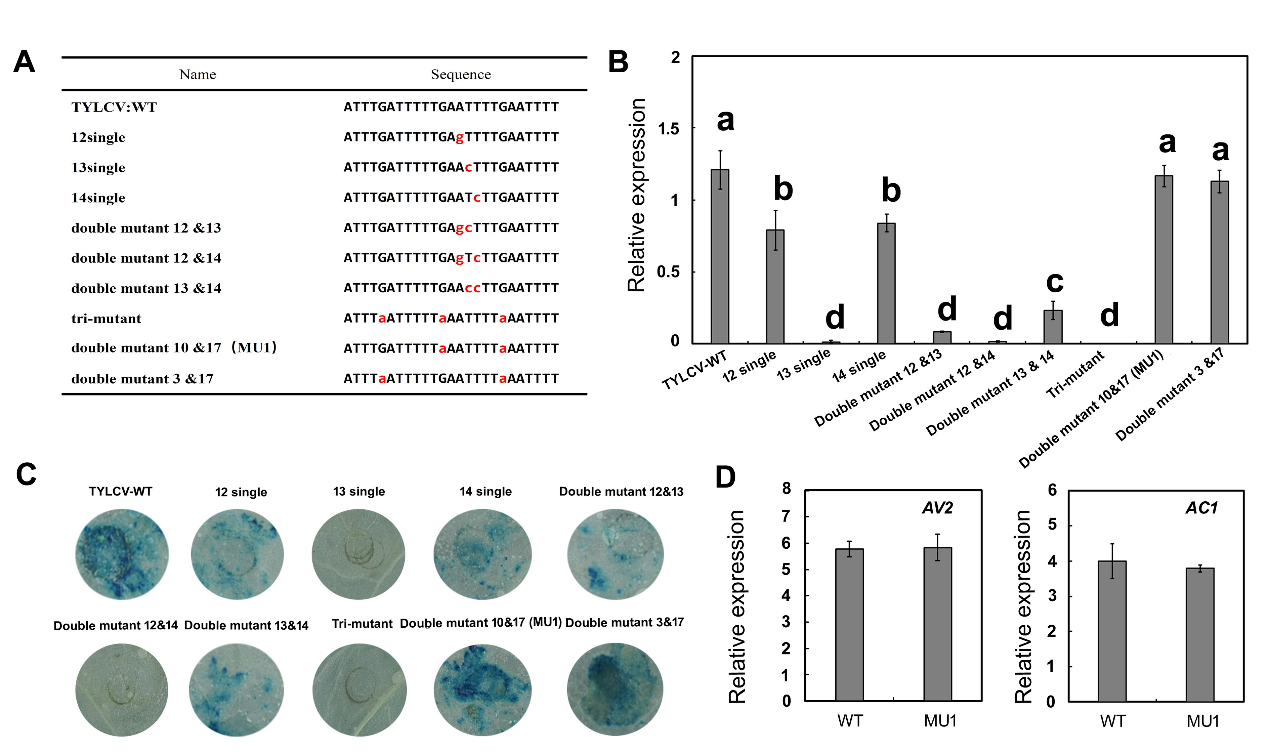


S6 Fig. The replication and promoter activities of different TYLCV mutants in *N. benthamiana*. (A) The TYLCV mutants generated. The mutanted nuceltides were shown with red-labeld lower letters. (B) The promoter activities of the mutants. The relative expression of *AC1* gene was determined by qRT-PCR calculated in relation to the *N. benthamiana* *EF1a* gene according to the ^Δ^Ct method (different letter, p<0.05). (C) The GUS staining of leaves inoculated by different IR mutants. The indicated IR mutants were fused with GUS reporter gene for plant transformation and the promoter activity analysis. (D) The relative expression of *AV2* and *AC1* genes in the wild type TYLCV and MU1-treated *N. benthamiana* plants. The expression value was measured by qRT-PCR and calculated in relation to the *EF1a* of *N. benthamiana* according to the ^Δ^Ct method. Error bars represented SD of three biological replicates. The RNA sample was extracted at 3 days after treatment.
