# Supplementary material for: Tomato yellow leaf curl virus intergenic siRNAs target a host long noncoding RNA to modulate disease symptoms
Source: PLoS Pathog. 2019 Jan 22;15(1):e1007534. doi: 10.1371/journal.ppat.1007534 (PMC6366713; doi:10.1371/journal.ppat.1007534)
Supplement: S1 Table — (DOCX) [file ppat.1007534.s007.docx]

Supporting Information

S1 Table. Oligonucleotides used in the study

| No | Name | Sequences (5' to 3’) | Purpose |
| --- | --- | --- | --- |
| 1 | JAGN1037F | CTTATACCTGGACACCTAATGGCT | qRT-PCR analysis for IR of TYLCV |
| 2 | JAGN1038R | AGTAACGTCTGTGGAACCCTCG |  |
| 3 | JAGN2126F | GGATTTCGTTGTATGTTAGC | qRT-PCR analysis for *AV2* of TYLCV, also for TYLCV quantitation |
| 4 | JAGN2127R | ATGATTATATCGCCTGGTC |  |
| 5 | JAGN6533 | GCGACCCACTCTTCAAGTTC | qRT-PCR analysis for *AC1* of TYLCV |
| 6 | JAGN6534 | AGGAGGTCAACAATCTGCCA |  |
| 7 | JAGN1494F | CATGTTGAAATGAATTGGTGTC | IR specific primers for reverse transcribed and RT-PCR |
| 8 | JAGN1495R | AAGACAAACTACTTGGGGAC |  |
| 9 | JAGN1538 | GACTCTAGACATGTTGAAATGAATTGGTGTCC | Insertion of IR into TRV2 vector |
| 10 | JAGN1539 | CATGGATCCGCAAGACAAACTACTTGGGGAC |  |
| 11 | JAGN1478R | CACATCAAAGCACACCTTCATCT | The 1^st^ round of 5’ RACE, and the reverse primer for amplifying the probe for *In situ* hybridization. |
| 12 | JAGN921R | CTTGATGACTTACGCCAATGACA | The 2^nd^ round of 5’ RACE and qRT-PCR of *SlLNR1* |
| 13 | JAGN920F | CCAGAAGAAATACTTGGAAGAAGC | The 1^st^ round of 3’ RACE and qRT-PCR of *SlLNR1* |
| 14 | JAGN2309F | AGATGAGTTGGTGGGCATAAGTG | The 2^nd^ round of 3’ RACE |
| 15 | JAGN2414F | AGCAGCAAATCCAGCGACCCGAG | To amplify the full transcript of *SlLNR1* |
| 16 | JAGN2415R | GTTGAATAATATTTAAGTTCACATCAGCA |  |
| 17 | JAGN1477F | AATCGAACGGCTCAAATCAAGTT | *SlLNR1* specific primer for reverse transcription of its anti-sense strand and RT-PCR, and the sense primer for amplifying the probe for *In situ* hybridization. |
| 18 | JAGN2225F | CGCGGATCCAGCAGCAAATCCAGCGACCCGAG | To amplify sense sequence of *SlLNR1* gene for transient expression in *N. benthamiana,* and the construct was also used for stable expression in tomato |
| 19 | JAGN2226R | CGGGGTACCGTTGAATAATATTTAAGTTCACATCAGCA |  |
| 20 | JAGN2227F | CGGGGTACCAGCAGCAAATCCAGCGACCCGAG | To amplify anti-sense sequence of *SlLNR1* gene for transient expression in *N. benthamiana* |
| 21 | JAGN2228R | CGCGGATCCGTTGAATAATATTTAAGTTCACATCAGCA |  |
| 22 | JAGN2359F | CATAAGCTTCATGTTGAAATGAATTGGTGTC | Insertion of IR into pBI101 vector |
| 23 | JAGN2360R | AACGGATCCAAGACAAACTACTTGGGGAC |  |
| 24 | JAGN2241__I miR-s | gaTTGATTTTTGAATTTTGAATTtctctcttttgtattcc | Insertion of siRNA(-2732-21) into pCAMBIA2301 vector. The vector for expressing siRNA(-2732-21) use *Arabidopsis* miR319a precursor gene as backbone and further ligated to pCAMBIA2301. |
| 25 | JAGN2242__II miR-a | gaAATTCAAAATTCAAAAATCAAtcaaagagaatcaatga |  |
| 26 | JAGN2243__III miR*s | gaAACTCAAAATTCATAAATCATtcacaggtcgtgatatg |  |
| 27 | JAGN2244__IV miR*a | gaATGATTTATGAATTTTGAGTTtctacatatatattcct |  |
| 28 | JAGN994 | AGAGGCCCTCAGACAAAC | qRT-PCR analysis of *EF1a* in *N. benthamiana* |
| 29 | JAGN995 | TAGGTCCAAAGGTCACAA |  |
| 30 | JAGN1301 | ACAAGATGGATGCTACCACC | qRT-PCR analysis of *EF1a* in *N.tabacum* |
| 31 | JAGN1302 | AACCAGAGATGGGGACAAAG |  |
| 32 | JAGN1015 | TGGTCGGAATGGGACAGAAG | qRT-PCR analysis of *actin* in tomato |
| 33 | JAGN1016 | CTCAGTCAGGAGAACAGGGT |  |
| 34 | JAGN1504 | GTTGGATCCAGCCCGAAACAATGAAAGAAAGT | RNAi construct of *SlLNR1* |
| 35 | JAGN1505 | GCTAAGCTTGAATTGGTACATTTCAGCTGGC |  |
| 36 | JAGN1506 | GAACTCGAGAATTGGTACATTTCAGCTGGC |  |
| 37 | JAGN1507 | CACGAGCTCAGCCCGAAACAATGAAAGAAAGT |  |
| 38 | JAGN2401 | CATAAGCTTCATGTTGAAATGAATTGGTGTC | Insertion of the sense strand of IR into pCAMBIA2301 vector |
| 39 | JAGN2402 | AACGAGCTCAAGACAAACTACTTGGGGAC |  |
| 40 | JAGN2375 | AACGAGCTCCATGTTGAAATGAATTGGTGTCC | Insertion of the anti-sense strand of IR into pCAMBIA2301 vector |
| 41 | JAGN2376 | GACTCTAGAAAGACAAACTACTTGGGGAC |  |
| 42 | JAGN2388 | GCTGATGGCGATGAATGAACACTG | 5' RACE Outer Primer for cleavage site validation |
| 43 | JAGN2389 | CGCGGATCCGAACACTGCGTTTGCTGGCTTTGATG | 5' RACE inner Primer for cleavage site validation |
| 44 | JAGN2580 | TCTAGGTATCACAATCTAAGGCGCTCTTCGTCC | 5' RACE gene specific primers |
| 45 | JAGN2581 | CAAGGAATCGAACGGCTCAAATCAAGTTTCCG |  |
| 46 | JAGN2582 | CGTTGAGAATTGGTACATTTCAGCTGGCCGAC |  |
| 47 | JAGN7048 | CCTCGCATTACCCTTACG | qRT-PCR analysis of GUS gene |
| 48 | JAGN7049 | TGACTGCCTCTTCGCTGT |  |
| 49 | JAGN2306 | TCTAAGCTTTCGTCCGCAACGTGAAGATCGTT | Insertion of the promoter of *SlLNR1* into PBI101 vector |
| 50 | JAGN2307 | TCAGGATCCAAATCGGATGAGGAAAGGGGATA |  |
| 51 | JAGN6179 | CGGAATTCCCACTATCTTCCTCTGC | Tylcv-xh-F |
| 52 | JAGN6180 | CGGAATTCCCCCTTTAATTTGAATGG | Tylcv-xh-R |
| 53 | JAGN6181 | CAATTCAAAATTCAAAACTCAAAAATCAAATCATTAAA | 12 single TYLCV mutant infectious clone construction |
| 54 | JAGN6187 | ATTTTTGAgTTTTGAATTTTGAATTGCAATGTACTTTT |  |
| 55 | JAGN6182 | CAATTCAAAATTCAAAGTTCAAAAATCAAATCATTAAA | 13 single TYLCV mutant infectious clone construction |
| 56 | JAGN6188 | ATTTTTGAAcTTTGAATTTTGAATTGCAATGTACTTTT |  |
| 57 | JAGN6183 | CAATTCAAAATTCAAGATTCAAAAATCAAATCATTAAA | 14 single TYLCV mutant infectious clone construction |
| 58 | JAGN6189 | ATTTTTGAATcTTGAATTTTGAATTGCAATGTACTTTT |  |
| 59 | JAGN6184 | CAATTCAAAATTCAAAgcTCAAAAATCAAATCATTAAA | Double mutant 12 & 13 TYLCV mutant infectious clone construction |
| 60 | JAGN6190 | ATTTTTGAgcTTTGAATTTTGAATTGCAATGTACTTTT |  |
| 61 | JAGN6185 | CAATTCAAAATTCAAgAcTCAAAAATCAAATCATTAAA | Double mutant 12 & 14 TYLCV mutant infectious clone construction |
| 62 | JAGN6191 | ATTTTTGAgTcTTGAATTTTGAATTGCAATGTACTTTT |  |
| 63 | JAGN6186 | CAATTCAAAATTCAAggTTCAAAAATCAAATCATTAAA | Double mutant 13 & 14 TYLCV mutant infectious clone construction |
| 64 | JAGN6192 | ATTTTTGAAccTTGAATTTTGAATTGCAATGTACTTTT |  |
| 65 | JAGN6546 | CAAAATTtAAAATTtAAAAATtAAATCATTAAAGCG | Tri-mutant TYLCV infectious clone construction |
| 66 | JAGN6547 | ATGATTTaATTTTTaAATTTTaAATTTTGAATTGCAATGT |  |
| 67 | JAGN6548 | CAAAATTtAAAATTtAAAAATCAAATCATTAAAGCG | TYLCV infectious clone (MU1) construction |
| 68 | JAGN6549 | ATGATTTGATTTTTaAATTTTaAATTTTGAATTGCAATGT |  |
| 69 | JAGN6552 | CAAAATTtAAAATTCAAAAATtAAATCATTAAAGCG | Double mutant 3 & 17 TYLCV mutant infectious clone construction |
| 70 | JAGN6553 | ATGATTTaATTTTTGAATTTTaAATTTTGAATTGCAATGT |  |
| 71 | TRV2 2b-F | ATGCACGAATTACTTAGGAAG | Determination of the presence of TRV2 |
| 72 | TRV2 2b-R | GGTAACCTTACTCACAGAAT |  |
| 73 | TRV1 Rep-F | ATCTCAAGTTGATTTGAGGTT | Determination of the presence of TRV1 |
| 74 | TRV1 Rep-R | TGATCTCTTTGCTTACATCGT |  |
| 75 | JAGN1899 | CAATCTAGACTCTGTAGCTCGTTGTTCTCGG | *SlLNR1* fragment chosen for VIGS |
| 76 | JAGN1900 | CAAGGATCCGACCAATTTCATCCATCAGAGGT |  |
| 77 | UBQ14-qF | CAACGCTCCATCTTGTCCTT | qRT-PCR primers for *UBQ14* of cotton |
| 78 | UBQ14-qR | TGATCGTCTTTCCCGTAAGC |  |
