# Supplementary material for: Tomato yellow leaf curl virus intergenic siRNAs target a host long noncoding RNA to modulate disease symptoms
Source: PLoS Pathog. 2019 Jan 22;15(1):e1007534. doi: 10.1371/journal.ppat.1007534 (PMC6366713; doi:10.1371/journal.ppat.1007534)
Supplement: S2 Table — (DOCX) [file ppat.1007534.s008.docx]

Supporting Information

S2 Table. The constructs used

| No | Plasmid Name | Sources | Construct and purpose |
| --- | --- | --- | --- |
| 1 | pTRV: IR | pTRV2 | TYLCV IR was inserted into pTRV2 by *Xba* I/*BamH* I for IR expression in plant. |
| 3 | pTRV: 4TR | pTRV2 | Containing 4×25-nt sequence of aaaattcaaaattcaaaaatcaaat by directly synthesized and was inserted into pTRV2 by *EcoR* I/*BamH* I for expression of siRNA(-2732-21) |
| 4 | pCAMBIA2301: *SlLNR1*(+) | pCAMBIA2301 | The sense *SlLNR1* was inserted into pCAMBIA2301 by *BamH* I/*Kpn* I for transient expression in *N. benthamiana* and over expression in tomato. |
| 5 | pCAMBIA2301:siRNA | pCAMBIA2301 | The artificial siRNA(-2732-21) was inserted into pCAMBIA2301 by *Kpn* I/*Sac* I for transient expression in *N. benthamiana* |
| 6 | pGEM-T-*SlLNR1* | pGEM-T | Containing partial fragment of *SlLNR1* for *in situ* hybridization |
| 7 | 12single | pBINPLUS | 12 single TYLCV mutant infectious clone construction |
| 8 | 13single | pBINPLUS | 13 single TYLCV mutant infectious clone construction |
| 9 | 14single | pBINPLUS | 14 single TYLCV mutant infectious clone construction |
| 10 | Double mutant 12 &13 | pBINPLUS | Double mutant 12 & 13 TYLCV mutant infectious clone construction |
| 11 | Double mutant 12 &14 | pBINPLUS | Double mutant 12 & 14 TYLCV mutant infectious clone construction |
| 12 | Double mutant 13 &14 | pBINPLUS | Double mutant 13 & 14 TYLCV mutant infectious clone construction |
| 13 | Tri-mutant | pBINPLUS | Tri-mutant TYLCV infectious clone construction |
| 14 | Double mutant 10 &17（MU1） | pBINPLUS | TYLCV infectious clone (MU1) construction |
| 15 | Double mutant 3 &17 | pBINPLUS | Double mutant 3 & 17 TYLCV mutant infectious clone construction |
| 16 | Pro-*SlLNR1(R/S)*:GUS | pBI101 | The promoters of *SlLNR1* (for the sense transcript) in both the CLN2777A (R) and JS-CT-9210 (S) were inserted into pBI101 by *Xba* I/*BamH* I to verify its promoter activity |
| 17 | pTRV: *SlLNR1* | pTRV2 | A 359-bp of *SlLNR1* fragment was inserted into pTRV2 by *Xba* I/*BamH* I for silencing *SlLNR1*. |
| 18 | pCAMBIA2301: *SlLNR1*(-) | pCAMBIA2301 | A 297-bp of *SlLNR1* fragment was ligated in opposite orientation on either side of the intron derived from hurricane by *Hind*Ⅲ /*BamH* I and *Xho* I/*Sac* I, the hurricane-RNAi was ligated to the expression vector pCAMBIA2301 by *BamH* I/*Sac* I. |
